# Conservation Potential Trough In Vitro Regeneration of Two Threatened Medicinal Plants *Ungernia sewertzowii* and *U. victoris*

**DOI:** 10.3390/plants13141966

**Published:** 2024-07-18

**Authors:** Feruza Usmanovna Mustafina, Hanifabonu Kobul kizi Juraeva, Dilafruz Nematilla kizi Jamalova, Abbos Tulkin ogli Hazratov, Ayimxan Jalgasbaevna Janabaeva, Hoe Jin Kim, Chae Sun Na, Min Sung Lee, Yu Jin Oh, Komiljon Sharobiddinovich Tojibaev, Sodikjon Kholiknazarovich Abdinazarov

**Affiliations:** 1 Tashkent Botanical Garden Named after Acad. F.N. Rusanov of the Institute of Botany of the Academy of Sciences of the Republic of Uzbekistan, Tashkent 100140, Uzbekistan; mustafinaferuza@yahoo.com (F.U.M.); hanifabonujurayeva@gmail.com (H.K.k.J.); xazratovabbos65@gmail.com (A.T.o.H.); ayimxan0105@gmail.com (A.J.J.); botanika-t@mail.ru (S.K.A.); 2 Institute of Botany of the Academy of Sciences of the Republic of Uzbekistan, Tashkent 100125, Uzbekistan; dilafruz.bel.91@mail.ru (D.N.k.J.); ktojibaev@mail.ru (K.S.T.); 3 Baekdudaegan National Arboretum, Bonghwa 36209, Republic of Korea; chaesun.na@koagi.or.kr (C.S.N.); next9101@koagi.or.kr (M.S.L.); oyj0705@koagi.or.kr (Y.J.O.)

**Keywords:** medicinal plants, in vitro propagation, conservation, endangered plants, alkaloids, DPPH, ABTS

## Abstract

*Ungernia sewertzowii* (US) and *U. victoris* (UV) are medicinal plants and sources of biologically active compounds for pharmaceutical needs. The leaves of US contain 0.29–0.81% sum of alkaloids with a predominance of lycorine, which is 0.04–0.46% in leaves and 0.15–0.38% in bulbs. Lycorine is used to treat acute and chronic bronchitis. The leaves of UV contain 0.27–0.71% sum of alkaloids with a predominance of galanthamine—0.13–1.15%. Galanthamine is used to treat mild-to-moderate dementia (Alzheimer’s disease). The natural populations of US and UV are in danger as sources of income for local people. To resolve this problem, two protocols for microclonal propagation were developed to replace natural raw materials with in vitro regenerated plants. Callusogenesis of US and UV was induced on Murashige and Skoog (MS) nutrient media with 2.4D (0.5 mg/L) in combination with BAP (0.5 mg/L), Kin (0.5 mg/L), or Zea (0.5 mg/L). Direct (for US) and indirect (for US and UN) organogenesis were observed on MS with BAP (0.5 mg/L) or Kin (0.5 mg/L) in combination with IAA (0.5 mg/L) or NAA (0.5 mg/L). Direct organogenesis resulted in 3–5 bulbs of US on one explant; indirect organogenesis resulted in up to 100–150 bulbs of US and UV on one explant within 6 months, or five to six subcultures after transferring the callus to the nutrient medium. The tissue cultures of US and UV were characterized by very low data on antioxidant activity based on IC50 values for DPPH and ABTS radical scavenging activities, whereas in vitro regenerated plants (leaves and bulbs) had higher data. We concluded that in vitro regenerated plants are valuable sources of lycorine and galanthamine, which allow the protection of the natural populations of these two species from extinction.

## 1. Introduction

The genus *Ungernia* Bunge (Amaryllidaceae J.St.-Hil.) is represented by six species native to Central Asia, of which four species grow in Uzbekistan [1,2]. Two of these species are valuable medicinal plants distributed in mountainous regions [3,4,5]: *U. sewertzowii* (Regel) B.Fedtsch., Sewerzow’s ungernia, and *U. victoris* Vved. ex Artjush., Viktor’s ungernia.

**Figure 1:**
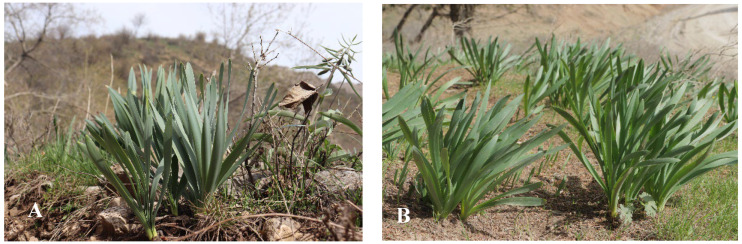
(**A**). *Ungernia sewertzowii*. Adult plants. Western Tien Shan, Pskem range, Aksarsay river, vicinity of Nanay village. 6 May 2022. Photo by Mustafina F.U. (**B**). *Ungernia victoris*. Adult plants. Pamir-Alay, Gissar range, basin of the Sangardak river, right bank, vicinity to Sangardak village. 28 March 2022. Photo by Turdiev D.T.

*U. sewertzowii* (Regel) B.Fedtsch., Sewerzow’s ungernia (US), a perennial plant endemic to the Western Tien Shan, is distributed in the Parkent region of Tashkent province, Uzbekistan (Figure 1A). The area of distribution is mostly among stones in mountains, on shallow slopes at a level of 600 to 2800 masl. In submountain regions, this species is distributed among steppe vegetation; at higher levels, the species is found among trees and bushes. Usually, this species grows in small groups on stony and gravel slopes (https://planta-medica.uz (accessed on 9 July 2024)).

The share of the alkaloids of US varies depending on the region of the collection of plant material (the plants from Karjantau, Western Tien Shan, Uzbekistan part, and Burchmulla, Bostanlyk region, Uzbekistan, were comparatively studied) and the part of the plant [6]: the leaves contain from 0.29% (Burchmulla) up to 0.81% (Karjantau) of alkaloids; the bulbs contain from 0.25% (Burchmulla) to 1.86% (Karjantau) of alkaloids; and the roots contain from 1.44% (Burchmulla) to 2.15% (Karjantau) of alkaloids. The leaves of Sewerzow’s ungernia include important biologically active compounds with a predominance of lycorine (0.04% in leaves and 0.15% in bulbs from Burchmulla and up to 0.46% in leaves and 0.38% in bulbs from Karjantau). According to Smirnova et al. (1964) [7], US contains other biologically active compounds, such as pancratine (0.12% in leaves and 0.64% in bulbs), galanthamine (0.01% in leaves and 0.033% in bulbs), narwedine (0.03% in leaves and 0.01% in bulbs), hippeastrine (0.0008% in leaves and 0.0002% in bulbs), ungiminorine (0.0005% in leaves and 0.074% in bulbs), tazettine (0.0001% in leaves and 0.1% in bulbs), ungerine (0.043% in leaves and 0.089% in bulbs), and unsevine (0.016% only in roots). Lycorine is used to treat acute and chronic bronchitis; it has strong vomiting and expectorant effects. Lycorine is the most widely distributed Amaryllidaceae alkaloid with antiviral, antifungal, and anti-inflammatory properties [8]. Currently, lycorine hydrochloride is applied in medicine as an expectorant for chronic and acute inflammatory processes in the lungs and bronchi and for bronchial asthma; it is used in anticancer research as a novel anti-ovarian cancer agent [9,10]. Lycorine inhibits protein synthesis [11] and weakly inhibits acetylcholinesterase (AChE) and ascorbic acid biosynthesis [12,13,14].

The leaves of US are collected as raw material in April, when they reach their maximum development (30–35 cm in length). The collection of the leaves starts in the middle of April at the level of 600–1200 masl and at the end of April at the level of 1800–2700 masl [4,5].

Fresh leaves are cut into pieces of 2–3 cm on the day of collection from nature. The leaves are laid out in a thin layer on a tarpaulin and raked 2–3 times a day to speed up drying. The raw materials are dried for 4–5 days. Baked *U. sewertzowii* bulbs are used in folk medicine as a wound-healing agent; they are also applied to boils to cleanse pus.

*U. victoris* Vved. ex Artjush., Viktor’s ungernia (UV)—perennial plant, included in the Red Book of rare, threatened, and disappearing species of the flora of the Republic of Uzbekistan with status 2—a rare endemic of Central Asia growing on the slopes of the Gissar mountains of the Southwest Pamir-Alay with a strongly decreasing distribution area (Figure 1B). The main cause of the decrease in its populations is the intensive collection of the leaves as a valuable source of biologically active compounds (BAC), resulting in the depletion of the bulbs and slow regeneration of the plantations. The area of distribution of the plant is mostly on stony slopes ranging from 800 to 2700 masl [15] (https://planta-medica.uz (accessed on 9 July 2024)). In the foothills and middle mountain zone, this plant is confined to wheatgrass steppes; higher up, it is found in the belt of tree and shrub vegetation under the canopy of junipers, among thickets of bushes, as well as in fescue and fescue–wheatgrass steppes. Usually, UV grows in small groups, occupying fine-earth areas on gravelly and rocky slopes. At the old camps, this species often formed almost huge thickets. However, in spite of the procurement to collect this rare plant, UV populations are in catastrophic condition because of extensive collection by pharmaceutical organizations and local people.

The sum of alkaloids in *U. victoris* varies according to the place of collection and climatic conditions [16]. The total alkaloids in the leaves and bulbs were found to be 0.5–0.52%. The proportion of galanthamine in the alkaloid mixtures was around 0.13–0.15% in dried leaves [17]. About 10 alkaloids have been reported in this plant species: galanthamine, narwedine, lycorine, ungerine, ungeridine, hippeastrine, haemanthidine, tazettine, nortazettine, and hordenine [17,18]. Dried leaves of *U. victors* also contain other alkaloids such as lykorine (0.073%), hordenine (0.039%), tazettine (0.1%), pankratine (0.15%), and narwedine (0.0054%) [17].

The plants rich in galanthamine were historically used in folk medicine in the Caucasus. For the first time, galanthamine was extracted by Proskurnikova and Yakovleva in 1952 from *Galanthus woronowii* Losinsk [19]. In 1956, galanthamine was found by Bulgarian scientists Paskov and Ivanova-Bubeva in *Galanthus nivalis* var. gracilis [20]. The industrial production of galanthamine under the brand name Nivalin^®^ started in 1958. The first clinical research on galanthamine was started in East European countries as a potential preventive measure against poliomyelitis. In 1980, the first preclinical research was conducted against Alzheimer’s disease. In 1996, the Austrian pharmaceutical company Sanochemia Pharmazeutika started to produce medical preparations under the brand name Nivalin^®^. In 1997, this company developed an industrial method of galanthamine production, which considerably decreased the price of this compound (the price of the pure component was USD 40,000). The British company Shire Pharmaceuticals and the Belgian research organization Janssen Research Foundation for Alzheimer’s Diseaseruen developed and produced medical preparations under the brand name Reminyl^®^, including galanthamine [21,22,23].

The leaves of UV are collected as raw material when they reach 30–35 cm in length. Fresh leaves are cut into pieces 2–3 cm long and laid out in a thin layer for 4–5 days. Coarsely chopped and dried leaves are used as raw materials to obtain galanthamine hydrobromide. Bulbs with roots contain more galanthamine than leaves, but lycorine predominates in them. Currently, the leaves of UV serve as the main raw material source for galanthamine, produced in the form of galanthamine hydrobromide. Galanthamine hydrobromide is used for myasthenia gravis, progressive muscular dystrophy, motor and tactile disorders associated with neuritis, polyneuritis, and Alzheimer’s disease [24].

The protocol for the synthesis of lycorine hydrochloride with the use of dried leaves of *U. sewertzowii* as a raw material [25] and the methodology of galanthamine extraction from *U. victoris* leaves [26] were developed at the Institute of the Chemistry of Plant Substances (ICPS), named after acad. S.Yu. Yunusov of the Academy of Sciences of the Republic of Uzbekistan, and were implemented in industry. The main source of raw materials is natural populations. Although the *Ungernia* species we studied perfectly reproduce both by seeds and vegetatively by producing daughter bulbs, and seed propagation is one of the most effective ways of dispersing these species, the collection of plants from natural populations is a good source of income for local people and, therefore, the natural populations of *Ungernia* are under strong anthropogenic pressure and in danger.

The purpose of the present investigations is to develop protocols for in vitro propagation of two rare medicinal plants, *U. sewertzowii* and *U. victoris*, to replace the natural sources of biologically active components by in vitro tissue culture or/and regenerated plants to supply ICPS as a potential consumer with raw material at an industrial level and decrease anthropogenic pressure on natural populations (Figure 1A,B).

## 2. Materials and Methods

Plant material of *U. sewertzowii* and *U. victoris* was collected from different populations growing in mountains (Table 1).

### 2.1. Plant Material

*Ungernia sewertzowii* (Regel) B.Fedtsch., Sewerzow’s ungernia (US). The plant is perennial and endemic to the Western Tien Shan. The leaves are 4–6 in number, two-ranked, glaucous, linear, smooth, slightly crisped, subequal, 1.5–2 cm in width, and ca. 20 cm in length, slightly tapering at the tip. The leaves start growing early in February and completely wither at the end of May and the beginning of July, leaving only grayish scales on the surface of the soil. Lycorine is the compound of interest, mostly accumulating in the leaves in the middle of April. The flowering starts at the end of July and continues until the beginning of August. The (5)-7-12-flowered umbel is located at the end of the single (15)-20–40 cm long stalk. The flowers are of brick red color on unequal pedicels of 2–8 cm in length or up to 10 cm in length when fruiting, and bracts are long. The perianth leaflets are 20–25 mm in length and 4–5 mm in width, three times longer than the tube, narrowly lanceolate, acutish, and becoming purple when dry; the fruits are 2–2.5 cm in diameter, ripening in September–early October [1] (Figure 1A).

*Ungernia victoris* Vved. ex Artjush., Victor’s ungernia (UV). The plant is perennial and endemic to the Southwest Pamir-Alay. The leaves are 7–10 in number, two-ranked, glaucous, linear, smooth, slightly crisped, subequal, 2–3 cm in width, 20–25 cm in length, and blunt at the tip. The leaves start growing in February and completely wither at the end of May and beginning of July, leaving only grayish scales on the surface of the soil. Galanthamine is the compound of interest, mostly accumulating in the leaves in the middle of April. The flowering starts at the beginning of August. The (2)-4-7-floral umbel is located at the end of a 12–30 cm long stalk. The flowers are yellowish pink on almost equal pedicels of 2–8 cm in length or up to 10 cm in length when fruiting; bracts are long. The perianth leaflets are 20–25 mm in length and 4–6 mm in width, 2–3 times longer than the tube, narrowly lanceolate, acutish, yellowish, or finally yellowish rose, becoming rosy when dry; the fruits are 3–4 cm in diameter, ripening in September [1] (Figure 1B).

### 2.2. Methods

#### 2.2.1. Sterilization of Explant

The solutions of sodium hypochlorite (4–6%), hydrogen peroxide (2–15%), silver nitrate (0.01%), Tween20, ethanol (70%), solution “Belizna” with a concentration of sodium hypochlorite of 18%, sterilizing soap “Domestos”, fungicides difenoconazole (Score 250EC, 23.3% *v*/*v*), mancozeb and metalaxyl (Ridamill Gold, 64% *v*/*v* and 4% *v*/*v* appropriately), fludioxonil (Maxim, 9.3% *v*/*v*), propiconazole (Agrotilt, 25% *v*/*v*), antibiotics streptomycin, amoxicillin, and gentamicin in concentrations of 1–4 mL/l were tested for the development of the sterilization protocol. More than 30 protocols with various combinations and concentrations of sterilizing agents have been implemented to find the optimal one that decreases or eliminates fungal contamination.

The material contaminated with fungi was transferred for examination to the phytopathology laboratory. It was found that material was mainly affected by Penicillium or Aspergillus fungi and internal infection (personal communication), and the most effective measures to combat these diseases were the system fungicide propicanazole (Agrotilt, 25% *v*/*v*) and contact fungicide fludioxonil (Maxim, 9.3% *v*/*v*). Both drugs were diluted with distilled water in a proportion of 0.2% *v*/*v* and used for plant material and seed sterilization to reduce contamination of the material with fungal diseases. Nutrient media (MS and Vch) supplemented with the antibiotic streptomycin (0.2% *v*/*v*) resulted in up to 80% sterility. The antibiotic agents amoxicillin and gentamicin were also tested but with less effect. The Petri dishes and jars were autoclaved at 2 atm. and 126 °C for 20 min before use.

#### 2.2.2. Explant Sources

**Figure 2:**
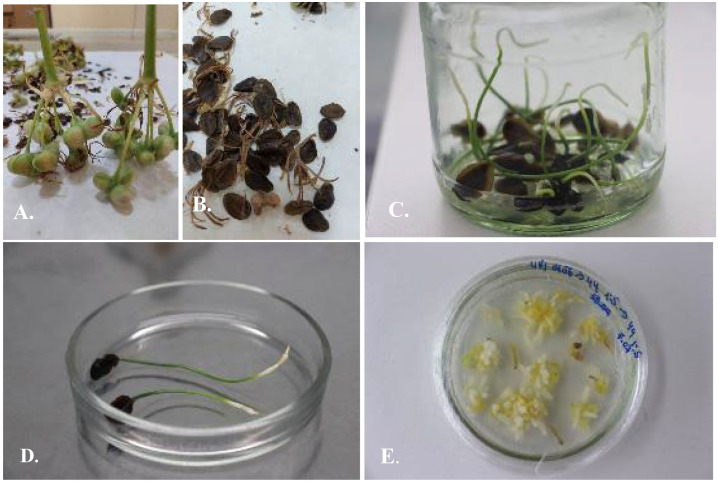
Stratification of the seeds of *Ungernia sewertzowii* and *Ungernia victoris.* (**A**). The seed pods. (**B**). The seeds with mechanical contamination. (**C**). Germinated seeds placed on a 50% MS nutrient medium in 0.5 L jars on shelves in the cultural room at +24 ± 2 °C and photoperiod 16/8 after stratification at −10 °C in the refrigerator for one month and at +5 °C for one month. (**D**). The segments of the germinated seeds (cotyledon, hypocotyl, and radicle) as the source of explants. (**E**). Callusogenesis on nutrient media M56 2.4D 0.5 mg/L + Kin 0.5 mg/L.

Different parts of the plants were used as explants for microclonal propagation:

1. Scales and bottoms of bulbs. Before work, the bulbs were cleaned from the soil and washed with tap water, and the outer scales were separated from the bulb itself. The inner scales were washed with running water and separated into individual scales. All scales were washed with running water and placed for 90 s in 70% ethanol, washed with distilled water, and soaked for 40 min in a 4% sodium hypochlorite solution. The scales were thoroughly washed with distilled water and cut into pieces of 5 × 5 mm. Pieces of scales were transferred to a solid nutrient medium with a cutting place. Before the explant pieces were transferred to a nutrient medium, they were soaked in a 4% hydrogen peroxide solution. Explants were placed in Petri dishes on 100% nutrient medium (25 mL) containing sucrose 30 g/L, agar 7 g/L, streptomycin 0.2% *v*/*v*, and phytohormones.

2. Seeds. Freshly collected seeds were placed in mesh bags of 20 × 30 cm in a freezer at a temperature of −10 °C for a period of more than 30 days. After 30 days of stratification at −10 °C, the seeds were separated from mechanical contamination, thoroughly washed under running water, placed in a 20% *v*/*v* solution of sterilizing soap “Domestos” with constant stirring at 150 rpm on a shaker, washed with distilled water, placed for 90 s in 70% ethanol, washed with distilled water, placed for 20 min in a 4% sodium hypochlorite solution, then washed again with distilled water. Twenty-five to thirty sterilized seeds were placed in Petri dishes on a 25% ready-made MS nutrient medium (25 mL) containing sucrose 7.5 g/L, agar 7 g/L, and streptomycin 0.2% *v*/*v* without phytohormones. Petri dishes with seeds were hermetically wrapped with elastic stretch film and placed in a refrigerator at a temperature of +5 °C for a period of 1–4 weeks in the dark. Then, the seeds were transferred to 25 mL of 50% ready-made MS nutrient media in 0.5 L sterile jars containing sucrose 15 g/L and agar 7 g/L without antibiotics and phytohormones. The jars with the seeds were allocated in a culture room at +24 ± 2 °C with photoperiod 16/8. The segments 0.5 mm in length of the germinated seeds (cotyledon, hypocotyl, and radicle) were used as the source of explants (Figure 2).

#### 2.2.3. Nutrient Media

The ready-made nutrient media from Duchefa Biochemie B.V (https://www.duchefa-biochemie.com) according to Murashige and Skoog (1962) (MS), Chu et al. (1975) (N6) [27,28,29], Gamborg et al. (1968) (B5) [30], and McCown Woody Plant Medium (Lloyd G. and McCown, 1980) (WPM) [31] were used. The nutrient medium by Vollosovich (1979) (Vch) [32,33,34,35] was prepared with reagents and biochemicals from Duchefa Biochemie B.V (https://www.duchefa-biochemie.com). Antibiotic streptomycin was added to the nutrient media in a concentration of 0.2% *v*/*v* only at the initial stages of in vitro cultivation, where bulb scales were used as explants, and to 25% MS media for seed stratification; later on, antibiotics were not used in the content of the nutrient media as the callus and regenerated plants were free of contamination. When optimizing the nutrient media, various concentrations of sucrose were tested (10–35%), and the most effective results with 30% sucrose were observed. Nutrient media with agar 7 g/L, sucrose 30 g/L, and streptomycin 0.2% *v*/*v* (at initial stages of in vitro cultivation with bulb scales as an explant and in 25% MS media for seed stratification) were autoclaved at 2 atm. and 126 °C for 20 min. In total, 25 ml of nutrient media were poured into each Petri dish preliminarily autoclaved at 2 atm. and 126 °C for 20 min.

#### 2.2.4. Phytohormones and Compositions

Stock solutions of auxins and cytokinins were prepared at a concentration of 1 mg/mL in a volume of 10 mL. The auxins 2,4-dichlorophenoxyacetic acid (2,4D), indole-3-acetic acid (IAA), α–naphthyl acetic acid (NAA), and indole-3-butyric acid (IBA) were dissolved in 10 mL of 70% ethyl alcohol. Cytokinins 6-benzylaminopurine (BAP) and kinetin (Kin) were dissolved in a small amount of 1 N alkali or acid solution and then diluted with distilled water up to 10 mL. More than 183 combinations of phytohormones with different concentrations were studied. Each combination was tested in at least three repetitions; in practice, up to 20–30 repetitions. We used a shortage from MS when defining one of the 183 media as M1–M183 and a shortage from Vch when defining one of the 183 media as V1–V183.

#### 2.2.5. Extraction

The samples of callus, in vitro regenerated plants, the plants introduced to the conditions of the Tashkent Botanical Garden, and the plants from different populations in the natural habitats of US and UV were studied. The samples were dried in the “Binder” (Tuttlingen, Germany) incubator at +50 ± C for three days. The dried and powdered samples of US and UV were successively extracted using 99% methanol with a solid-to-solvent ratio of 1:20 (*w*/*v*) at room temperature (2 times at 3-day intervals, totaling 6 days). The extracts were filtered through filter paper (No. 2, Whatman Co., Maidstone, Kent, UK) and evaporated at +40 °C to dryness using a rotary evaporator (Eyela N-1300, Tokyo Rikakikai Co., Ltd., Tokyo, Japan). The extracts were then dissolved in dimethyl sulfoxide (DMSO) and stored at −20 °C until used in subsequent experiments.

#### 2.2.6. DPPH Free Radical Scavenging Assay

The DPPH free radical scavenging assay was developed according to the methodology proposed by Shah et al. (2019), [6] with slight modifications, using 2,2-diphenyl-1-picrylhydryl free radical (DPPH) (Sigma Aldrich, St. Louis, MO, USA). A total of 200 µL of 150 µM DPPH in methanol was added to the samples, as much as 20 µL with various concentrations in a 96-well microplate. Ascorbic acid was used as a standard; all measurements were performed in triplicate. After 30 min, absorbance was determined at 517 nm using a UV spectrophotometer (Molecular Devices, San Jose, CA, USA).

#### 2.2.7. ABTS Free Radical Scavenging Assay

2,2-Azinobis (3-ethylbenzothiazoline)-6-sulphonic acid (ABTS) (Sigma Aldrich, St. Louis, MO, USA) assay was carried out according to the previously reported method [36]. The ABTS solution was prepared by mixing 7 mM ABTS and 2.45 mM potassium persulfate (final concentration) and then incubated in the dark at room temperature for 16 h. ABTS solution was diluted with distilled water for the absorbance to reach 0.70 ± 0.02 at 740 nm. Ten microliters of different concentrations of extract were added to 190 µL of ABTS solution. Antioxidant activity measurements were carried out in triplicate. Ascorbic acid was taken as a standard.

## 3. Result and Discussion

### 3.1. Use of Scales and Bottom of the Bulbs as the Source of Explants

The plants of US and UV for in vitro propagation were collected in 2021–2022 during the expeditions to the Tashkent, Jizzakh, and Surkhandarya regions of Uzbekistan (Figure 1A,B). Some of the bulbs were used as the source of explants, and some were planted in the experimental area of the Tashkent Botanical Garden. The bulbs of US are elongate-oblong in shape, 5–7 cm in diameter, and with mostly coal-black membranous coats predominantly produced at the neck. The UV bulbs are ovoid in shape, 4–7 cm in diameter, and with brown membranous coats more or less produced at the neck. The bulbs from nature populations and the Botanical Garden did not vary in shape, size, and structure.

The bulb’s scales and bottom were separated, and each scale was sterilized by washing with running water for 20 min, keeping in 70% ethanol for 90 s, then repeatedly washing under running water, keeping in a 4% sodium hypochlorite solution for 40 min, and washing in sterile distilled water in a laminar flow box. The bulb scales were cut into pieces of 5 × 5 mm and transferred to a solid nutrient medium with a cutting place. Before transferring to solid media, the explant pieces were soaked in a 4% hydrogen peroxide solution. When a silver nitrate solution was used in the sterilization protocol, most scales turned brown. That is why the silver nitrate solution was no longer used for the sterilization of *Ungernia* bulb scale explants. The use of fungicides did not affect the sterility of the explants, and therefore, the fungicides were removed from the sterilization protocol. The use of 4% sodium hypochlorite made it possible to obtain sterile and viable explants. When the concentration of sodium hypochlorite decreased to 2–3%, sterility of the explants was not achieved; when the concentration was higher than 4%, necrosis was observed, and the explants became brown. The most efficient protocol included the use of 70% ethanol for 90 s, then a 4% sodium hypochlorite solution for 40 min, then a 4% hydrogen peroxide solution for 2–3 s, and washing two to three times with distilled water at all steps. The implementation of this protocol resulted in 90% of viable explants.

#### Selection of Nutrient Medium

No changes in the explants’ structure were detected on N6, B5, and WPM media. Nutrient media MS, N6, B5, WPM, and Vch were tested with the bulb scales of *U. sewertzowii* and *U. victoris* as explants. Callusogenesis, indirect organogenesis, and direct/indirect somatic embryogenesis on MS and Vch were induced on bulb scales when tested with up to 183 phytohormone combinations, but none of the adult plants resulted from the observed processes. Callus induction was observed on the segments of bulb scales of US and UV on MS nutrient media, and somatic embryos were determined on Vch nutrient media.

We observed the following processes on the segments of the bulb scales of US and UV on MS:

1. Callusogenesis on the bulb scales of US on MS with phytohormones M39 NAA 2.0 mg/L, M55 2.4D 0.5 mg/L, M60 2.4D 1.0 mg/L + Kin 0.5 mg/L, and M56 2.4D 0.5 mg/L + Kin 0.5 mg/L and weak callus formation or its absence for UV with phytohormone 2.4D 2.0 mg/L and combination of phytohormones 2.4D 2.0–4.0 mg/L + BAP 0.5–4.0 mg/L.
2. Direct somatic embryogenesis on the bulb scales of US on MS with phytohormones M40 NAA 2.0 mg/L + BAP 0.5 mg/L and M56 2.4D 0.5 mg/L + Kin 0.5 mg/L.
3. Indirect somatic embryogenesis on the bulb scales of US on MS with phytohormones M56 2.4D 0.5 mg/L + Kin 0.5 mg/L.
4. No somatic embryogenesis was observed for UV on MS media.

The following processes on the segments of the bulb scales of US and UV were observed on the Vch nutrient medium:

1. Callusogenesis on the bulb scales of US on Vch with phytohormones V68 IAA 0.5 mg/L + Kin 0.5 mg/L, V17 IAA 0.5 mg/L + BAP 0.5 mg/L, and V56 2.4D 0.5 mg/L + Kin 0.5 mg/L.
2. Callusogenesis on the bulb scales of UV on Vch with phytohormones V56 2.4D 0.5 mg/L + Kin 0.5 mg/L, V5 2.4D 0.5 mg/L + BAP 0.5 mg/L, and V44 NAA 0.5 mg/L + Kin 0.5 mg/L.
3. Direct somatic embryogenesis on the bulb scales of US on Vch with phytohormones V29 Kin 1.0 mg/L, V68 IAA 0.5 mg/L + Kin 0.5 mg/L, V56 2.4D 0.5 mg/L + Kin 0.5 mg/L, V4 2.4D 0.5 mg/L, V16 IAA 0.5 mg/L, V81 2.4D 0.5 mg/L + Kin 5.0 mg/L, V83 IAA 0.5 mg/L + BAP 5.0 mg/L, V84 2.4D 0.5 mg/L + BAP 5.0 mg/L, and V87 2.4D 0.5 mg/L + Zea 0.5 mg/L.
4. Direct somatic embryogenesis on the bulb scales of UV with phytohormones V16 IAA 0.5 mg/L and V4 2.4D 0.5 mg/L.
5. Indirect somatic embryogenesis was not observed for US and UV on Vch media.

### 3.2. Using of Segments of Germinated Seeds as the Source of Explants

In the present study, it was revealed that the seeds of *Ungernia* species have a chilling obligation for germination. Stratification is necessary to end dormancy and hasten the germination of dormant seeds of US and UV. The segments of germinated seeds were used as an explant source for in vitro propagation of the studied species: hypocotyl, cotyledon, and radicle (Table 2).

### 3.3. Selection of Nutrient Media

**Figure 3:**
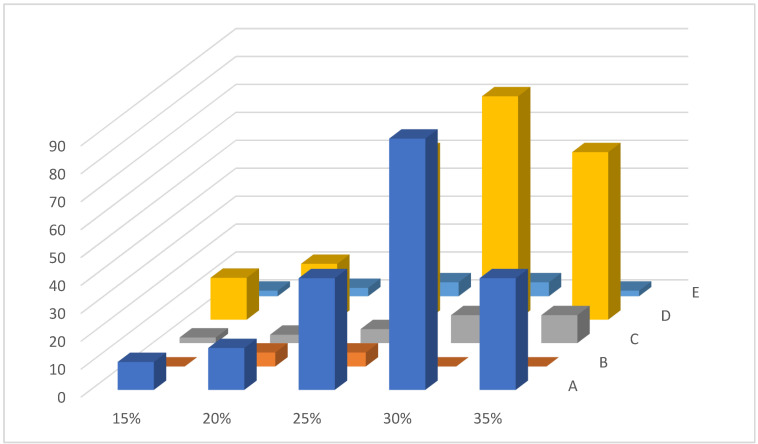
*Ungernia sewertzowii*. Callusogenesis on different nutrient media with various concentrations of sucrose. Source of explants—segments of the germinated seeds (hypocotyl, cotyledon, radicle). Nutrient media: (A) Murashige and Skoog [28], (B) Chu et al. [27], (C) Gamborg et al. [30], (D) by Vollosovich et al. [35], and (E) WPM [31].

Almost 90% of the explants showed changes in their structure on MS media with a sucrose concentration of 30% (Figure 3). No changes were observed on N6, B5, and WPM nutrient media. The concentration of sucrose has a critical significance: a low concentration did not affect the development of calluses while increasing the concentration resulted in the abnormal development of calluses for US and UV.

### 3.4. Protocol of Micropropagation of U. sewertzowii

In vitro propagation of *U. sewertzowii* was achieved by two approaches: direct and indirect organogenesis.

#### 3.4.1. Callusogenesis of *U. sewertzowi*

**Figure 4:**
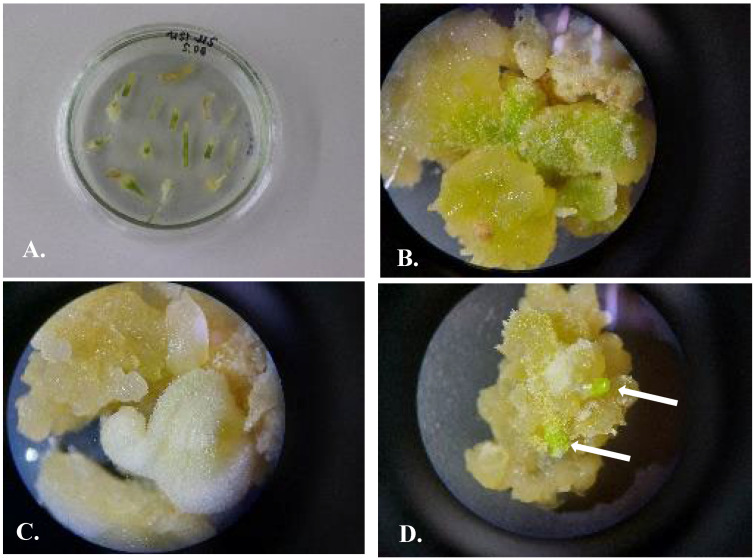
*Ungernia sewertzowii*. Callusogenesis on nutrient media by Murashige and Skoog (1962) [28] with phytohormones M5 2.4D 0.5 mg/L + BAP 0.5 mg/L. (**A**). Three weeks after placing the explants on nutrient media. (**B**,**C**). Callus after first subculture with green spots—rudiments of buds. (**D**). Callus after second subcultures with green buds (showed with arrows).

Callusogenesis of US was induced on MS nutrient media with the composition of phytohormones M5 2.4D 0.5 mg/L + BAP 0.5 mg/L (Figure 4), M56 2.4D 0.5 mg/L +Kin 0.5 mg/L, and M87 2.4D 0.5 mg/L + Zea 0.5 mg/L. The first subculture for proliferation was on the same nutrient media as used for inducing the callus: M5 2.4D 0.5 mg/L + BAP 0.5 mg/L, M56 2.4D 0.5 mg/L +Kin 0.5 mg/L, and M87 2.4D 0.5 mg/L + Zea 0.5 mg/L. The second subculture for the proliferation of the callus was on nutrient medium M56 2.4D 0.5 mg/L + Kin 0.5 mg/L. Intensive callus induction and proliferation were observed on the basal part of the hypocotyl and cotyledon for 95 ± 2% of explants, bright yellow in color, later becoming green. After the first and second subcultures to the new media, the development of buds was observed; within a short period of 1–2 weeks, the buds developed into microbulbs.

#### 3.4.2. Direct Organogenesis

For *U. sewertzowii*, direct organogenesis was induced on MS nutrient media with the combinations of the phytohormones M17 IAA 0.5 mg/L + BAP 0.5 mg/L, M32 NAA 0.5 mg/L + BAP 0.5 mg/L, M44 NAA 0.5 mg/L + Kin 0.5 mg/L, and M68 IAA 0.5 mg/L + Kin 0.5 mg/L. When the explants (cotyledon, hypocotyl, and radicle) were placed on a 100% MS nutrient medium with these combinations of phytohormones, direct organogenesis without callus induction was observed; in the case when callus was placed on a 100% MS nutrient medium, the development of indirect organogenesis was observed.

The first subculture was on the same media: M17 IAA 0.5 mg/L + BAP 0.5 mg/L, M32 NAA 0.5 mg/L + BAP 0.5 mg/L, M44 NAA 0.5 mg/L + Kin 0.5 mg/L, and M68 IAA 0.5 mg/L + Kin 0.5 mg/L. The second subculture for proliferation of the microbulbs was on 100% MS nutrient media with the ratio of auxin to cytokinin as 5:1: M17 IAA 2.5 mg/L + BAP 0.5 mg/L, M32 NAA 2.5 mg/L + BAP 0.5 mg/L, M44 NAA 2.5 mg/L + Kin 0.5 mg/L, and M68 IAA 2.5 mg/L + Kin 0.5 mg/L. The third subculture was on 50% MS nutrient media to stimulate the development of the root system: NAA 2.5 mg/L + BAP 0.5 mg/L + TDZ 0.3 mg/L. Further subcultures were on 50% MS nutrient media with NAA 0.5 mg/L, which stimulated the development of the root system and prepared regenerated plants for adapting to the soil.

Direct organogenesis allows for 3–5 bulbs on one explant, while indirect organogenesis allows for 100–150 well-developed plants on one explant ready for soil adaptation within 6 months after five subcultures. The morphogenic response to the composition of phytohormones in MS nutrient media was identical in direct and indirect organogenesis. For *U. sewertzowii*, the period from inducing in vitro culture up to adaptation to soil took 5–6 months.

M17 IAA 0.5 mg/L + BAP 0.5 mg/L. Callus formation was not observed on this nutrient medium. However, 2–3 microbulbs with a poorly developed root system or without roots were formed directly on each explant without callusogenesis.

M32 NAA 0.5 mg/L + BAP 0.5 mg/L. Callusogenesis was observed in 10 ± 2% of explants; the callus was light yellow. Well-developed microbulbs were formed, resulting from direct organogenesis, while the root system was poorly developed or absent. The formation of 2–3 microbulbs on one explant was observed on a number of explants.

M44 NAA 0.5 mg/L + Kin 0.5 mg/L. Callusogenesis was observed on 40 ± 2% light yellow explants. This combination of phytohormones stimulated the development of a root system. Small microbulbs were observed directly on explants or on the callus; the root system with microhairs was intensively formed. The direct formation of 2–3 microbulbs was observed on each explant.

M68 IAA 0.5 mg/L + Kin 0.5 mg/L. Weak callus formation was observed on 10 ± 2% of explants, and the callus was light yellow. The direct formation of comparatively well-formed 2–3 microbulbs was observed on each explant.

Among the tested 183 combinations, the most optimal phytohormones for callusogenesis were M5 2.4D 0.5 mg/L + BAP 0.5 mg/L, M56 2.4D 0.5 mg/L + Kin 0.5 mg/L, and M87 2.4D 0.5 mg/L + Zea 0.5 mg/L. Nutrient media with compositions of phytohormones M68 IAA 0.5 mg/L + Kin 0.5 mg/L, M32 NAA 0.5 mg/L + BAP 0.5 mg/L, and M17 IAA 0.5 mg/L + BAP 0.5 mg/L stimulated the development of the buds. On the nutrient medium M44 NAA 0.5 mg/L + Kin 0.5 mg/L, the development of the microbulbs was also observed but in a very small number.

#### 3.4.3. Indirect Organogenesis

When the callus was transferred to a 100% MS nutrient medium, the development of microbulbs was observed on the following combinations of phytohormones: M17 IAA 0.5 mg/L + BAP 0.5 mg/L, M32 NAA 0.5 mg/L + BAP 0.5 mg/L, M44 NAA 0.5 mg/L + Kin 0.5 mg/L, and M68 IAA 0.5 mg/L + Kin 0.5 mg/L. Placing explants (cotyledon, hypocotyl, and radicle) directly on nutrient media with combinations of these phytohormones stimulated direct organogenesis, but the number of bulbs was significantly less compared to indirect organogenesis (Table 3).

The first subculture of the callus with microbulbs was conducted in the same nutrient medium and compositions of phytohormones: M17 IAA 0.5 mg/L + BAP 0.5 mg/L, M32 NAA 0.5 mg/L + BAP 0.5 mg/L, M44 NAA 0.5 mg/L + Kin 0.5 mg/L, and M68 IAA 0.5 mg/L + Kin 0.5 mg/L. The second subculture was on nutrient media with a ratio of auxin to cytokinin of 5:1: M17 IAA 2.5 mg/L + BAP 0.5 mg/L, M32 NAA 2.5 mg/L + BAP 0.5 mg/L, and M68 IAA 2.5 mg/L + Kin 0.5 mg/L. The development of the root system was stimulated by using a 100% MS nutrient medium with the combination of the phytohormones M44 NAA 2.5 mg/L + Kin 0.5 mg/L. The development of the root system was stimulated on 50% MS media with the phytohormones IAA 2.5 mg/L + BAP 0.5 mg/L + TDZ 0.3 mg/L. Further subcultures were on a 50% MS nutrient medium with NAA 0.5 mg/L to stimulate the development of the root system and prepare plants for adapting to the soil.

Indirect organogenesis resulted in the formation of one explant of up to 100–150 well-developed plants ready for soil adaptation within 6 months after five subcultures.

The development of microbulbs was stimulated on MS nutrient media M17 IAA 2.5 mg/L + BAP 0.5 mg/L, M32 NAA 2.5 mg/L + BAP 0.5 mg/L, and M68 IAA 2.5 mg/L + Kin 0.5 mg/L; the development of the root system was stimulated on M44 NAA 2.5 mg/L + Kin 0.5 mg/L.

Direct and indirect organogeneses were observed for US on an MS nutrient medium with the segments of the germinated seeds as explants; direct organogenesis allowed for 3–5 bulbs on one explant, and indirect organogenesis allowed 100–150 bulbs on one explant. The morphogenic response to the composition of phytohormones in MS nutrient media was identical in direct and indirect organogenesis. Well-developed plants were transferred to the soil for further adaptation. For *U. sewertzowii*, the period from the transfer of callus to the nutrient media M17, M32, M44, and M68 up to adaptation to soil took up to 6 months after five subcultures.

### 3.5. Protocol of Micropropagation of U. victoris

**Figure 5:**
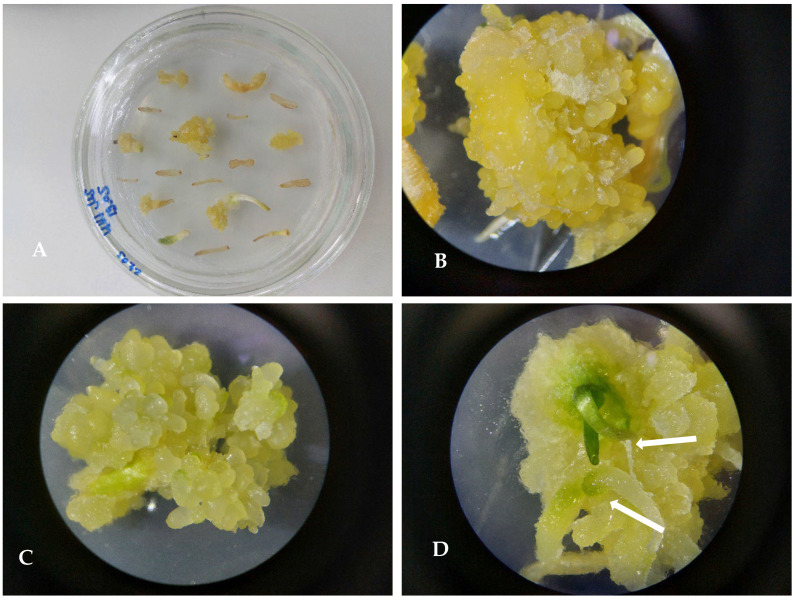
*Ungernia victoris*. Callusogenesis on nutrient media by Murashige and Skoog (1962) [28] with phytohormones M5 2.4D 0.5 mg/L + BAP 0.5 mg/L. (**A**). Three weeks after placing the explants on nutrient media. (**B**,**C**). Callus after the first subculture. (**D**). Callus after the second subculture with green buds (shown with arrows).

Callusogenesis of UV was induced on an MS nutrient medium with the compositions of phytohormones M5 2.4D 0.5 mg/L + BAP 0.5 mg/L (Figure 5), M56 2.4D 0.5 mg/L + Kin 0.5 mg/L, and M162 BAP 0.5 mg/L.

The first subculture of the callus was on an MS nutrient medium of the same composition and concentration of components as it was used for induction of in vitro culture: M5 2.4D 0.5 mg/L + BAP 0.5 mg/L, M56 2.4D 0.5 mg/L +Kin 0.5 mg/L, and M162 BAP 0.5 mg/L.

The second subculture of the callus for proliferation was on an MS nutrient medium M56 2.4D 0.5 mg/L + Kin 0.5 mg/L. Intensive callus induction and propagation were observed on the basal part of the hypocotyl and cotyledon for 80 ± 2% of explants; the callus was bright yellow, later becoming green. After the first and second subcultures, the development of buds was observed; within a short period of 1–2 weeks, the buds developed into microbulbs. Thidiazuron at a concentration of 0.01 mg/L stimulated the proliferation of the callus by 60–70%. Good results in callus proliferation were achieved by transferring from the medium M5 2.4D 0.5 mg/L + BAP 0.5 mg/L to the medium M56 2.4D 0.5 mg/L +Kin 0.5 mg/L.

#### 3.5.1. Indirect Organogenesis

**Figure 6:**
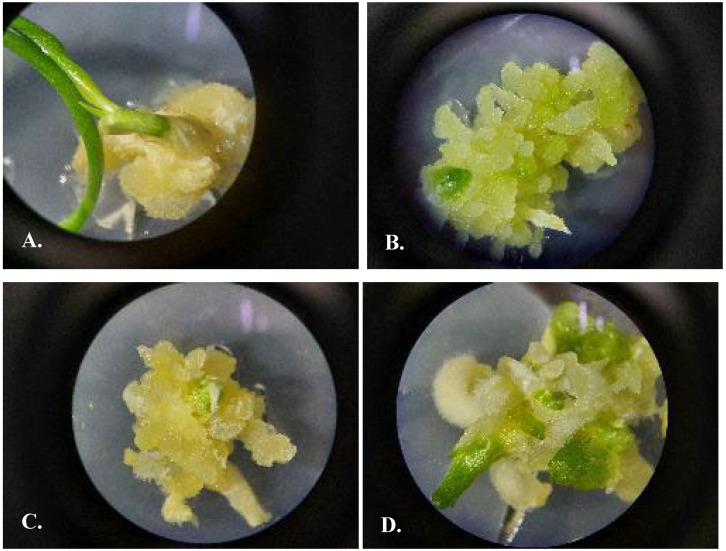
*Ungernia victoris*. Indirect organogenesis on nutrient media by Murashige and Skoog, 1962 [28]. (**A**). Callus induced on nutrient media M56 2.4D 0.5 mg/L + Kin 0.5 mg/L and transferred to nutrient media M17 IAA 0.5 mg/L + BAP 0.5 mg/L. (**B**). Development of green buds after first subculture from media M17 to the same media. (**C**,**D**). The rudiments of roots and buds. Sources of explants: segments of the germinated seeds (hypocotyl, cotyledon, and radicle).

**Figure 7:**
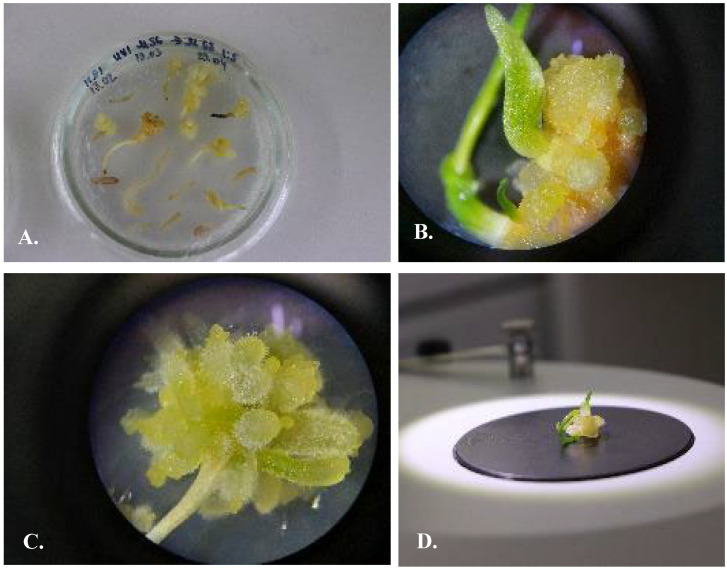
*Ungernia victoris*. Indirect organogenesis on nutrient media by Murashige and Skoog, 1962 [28]. (**A**). Callus induced on nutrient media M56 2.4D 0.5 mg/L + Kin 0.5 mg/L and transferred to nutrient media M68 IAA 0.5 mg/L + Kin 0.5 mg/L. (**B**). Development of green buds after first subculture from media M68 to the same media. (**C**). The rudiments of roots and buds. (**D**). Well-developed microbulbs after the third subculture.

For UV, the following combinations of phytohormones induced organogenesis: M17 IAA 0.5 mg/L + BAP 0.5 mg/L (Figure 6), M32 NAA 0.5 mg/L + BAP 0.5 mg/L, M44 NAA 0.5 mg/L + Kin 0.5 mg/L, and M68 IAA 0.5 mg/L + Kin 0.5 mg/L (Figure 7). When the callus was placed on a 100% MS nutrient medium, the development of indirect organogenesis was observed.

The first subculture of the callus was on the media with the same content and concentration of phytohormones: M17 IAA 0.5 mg/L + BAP 0.5 mg/L, M32 NAA 0.5 mg/L + BAP 0.5 mg/L, M44 NAA 0.5 mg/L + Kin 0.5 mg/L, and M68 IAA 0.5 mg/L + Kin 0.5 mg/L. The second subculture was on a 100% MS nutrient medium with a ratio of auxin to cytokinin of 5:1 for proliferation of the microbulbs: M17 IAA 2.5 mg/L + BAP 0.5 mg/L, M32 NAA 2.5 mg/L + BAP 0.5 mg/L, M44 NAA 2.5 mg/L + Kin 0.5 mg/L, and M68 IAA 2.5 mg/L + Kin 0.5 mg/L. The third subculture was on a 50% MS nutrient medium to stimulate the development of the root system: NAA 2.5 mg/L + BAP 0.5 mg/L + TDZ 0.3 mg/L. Further subcultures were on a 50% MS nutrient medium with NAA 0.5 mg/L, which stimulated the development of the root system and prepared regenerated plants for adaptation to the soil.

M17 IAA 0.5 mg/L + BAP 0.5 mg/L (Figure 6). Gemmogenesis, or the formation of microbulbs, was observed with a weakly developed root system. Microbulbs were characterized by well-developed leaves of up to 7–8 cm in length.

M32 NAA 0.5 mg/L + BAP 0.5 mg/L. Gemmorhizogenesis was observed; gemmogenesis was more intensive than rhizogenesis. The formation of up to 100–150 microbulbs was observed on one explant. In the initial stages, the callus was characterized by a light-yellow color, later turning to dark yellow or green. Dark yellow buds developed on the callus later became green, increased in length, and developed into microbulbs. The high rate of development of the leaves and the slow development of the root system were observed. Compared to M17, the root system was well-developed in this nutrient medium.

M44 NAA 0.5 mg/L + Kin 0.5 mg/L. As the callus transferred to this medium, we observed more intensive gemmorizogenesis than in the nutrient media M17, M32, and M68. Intensive development of root hairs and elongation of roots were observed. The formation of up to 100–200 microbulbs with a well-developed root system was noted on one explant. The bulbs transferred to this medium had developed roots, which allowed adaptation to the soil.

M68 IAA 0.5 mg/L + Kin 0.5 mg/L (Figure 7). This medium stimulated the intensive development of the microbulbs but the slow development of the root system. We observed 100–150 microbulbs on one explant.

**Figure 8:**
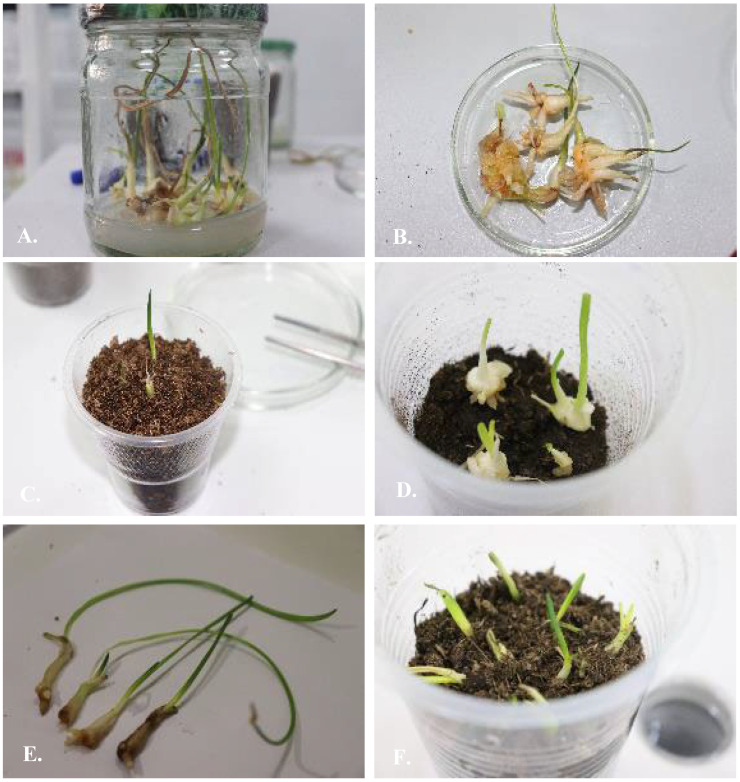
*Ungernia sewertzowii*. (**A**). Well-developed bulbs on hormone-free nutrient media by Murashige and Skoog, 1962 [28]. (**B**). Well-developed bulbs prepared for planting on soil. (**C**,**D**). Well-developed plants were transferred to the soil for adaptation. (**E**). *Ungernia victoris*. Well-developed bulbs with two leaves prepared for planting on soil. (**F**). Bulbs planted on soil for adaptation.

Indirect organogenesis observed for UV on an MS nutrient medium with the segments of germinating seeds as the explants resulted in the development of up to 100–150 bulbs on one explant (Table 4). The well-developed plants were transferred to the soil for further adaptation (Figure 8). For UV, the period from the transfer of callus to nutrient media M17, M32, M44, and M68 up to adaptation to soil took 5–6 months after five subcultures.

#### 3.5.2. DPPH and ABTS Analyses

The results of DPPH and ABTS analyses of the antioxidant activity of chemical components for US and UV are presented as Supplementary Material in Tables S12 and S13. The DPPH free radical scavenging assay was developed according to the methodology proposed by Shah et al., 2019 [6], with slight modifications, using a 2,2-diphenyl-1-picrylhydrylfree radical assay (DPPH) (Sigma Aldrich, USA) and a 2,2-Azinobis (3-ethylbenzothiazoline)-6-sulphonic acid radical assay (ABTS) (Sigma Aldrich, St. Louis, MO, USA) according to the previously reported method by Velioglu et al., 1998 [36].

For the *U. sewertzowii* US_3_1 samples collected in 2023 from the Aksarsay in the Tashkent region, the IC50 value of DPPH radical scavenging activity was 560.34 mg/mL, and the IC50 value of ABTS radical scavenging activity was 361.79 mg/mL, showing the third best result among US plant extracts collected in 2023 from nature. The US_3_2 sample collected from the same area had a good IC50 value of 622.00 mg/mL for DPPH radical scavenging activity and an IC50 value of 300.52 mg/mL for ABTS radical scavenging activity. The IC50 value of DPPH radical scavenging activity of the US_4_1 sample collected near the Bildirsay in the Tashkent region was the best at 433.10 mg/mL. The IC50 value of ABTS radical scavenging activity was 359.07 mg/mL, the highest among US extracts collected from nature. In addition, the IC50 value of the DPPH radical scavenging activity of the US_4_2 sample collected from the same area near the Beldersay in the Tashkent region was 442.00 mg/mL, and the IC50 value of the ABTS radical scavenging activity was 335.62 mg/mL, which is the best among US plant samples collected from nature. These results showed that the antioxidant activity of US plants varied depending on the region of collection. In addition, the results of analyses of the antioxidant activity of US extracts collected from the Aksarsay and Bildirsay and introduced in Tashkent Botanical Garden were excellent. More specifically, the IC50 value of the DPPH radical scavenging activity of the US_8 sample from the plant introduced in the Botanical Garden and collected in 2021 from the Aksarsay in the Tashkent region was 428.80 mg/mL, and the IC50 value of the ABTS radical scavenging activity was 297.02 mg/mL, showing that the US extract from the plants grown in the Botanical Garden had the best antioxidant activity compared to the plants collected from nature. The IC50 value of the DPPH radical scavenging activity of the US_9 sample of the plant from the Botanical Garden collected in 2021 in the Beldersay was 698.55 mg/mL, and the IC50 value of the ABTS radical scavenging activity was 457.37 mg/mL, showing good results among the US extracts from the plants grown in the Botanical Garden.

The IC50 values of the DPPH radical scavenging activity and the ABTS radical scavenging activity of US callus induced on two nutrient media, MS and Vch, did not vary. The IC50 values of the DPPH radical scavenging activity for callus developed on a Vch nutrient medium with a phytohormone composition of IAA 0.5 mg/L + Kin 0.5 mg/L, on an MS nutrient medium with a phytohormone composition of M56 2.4D 0.5 mg/L + Kin 0.5 mg/L, and on a Vch nutrient medium with a phytohormone composition of 2.4D 0.5 mg/L + Kin 1.0 mg/L were identical.

The regenerated plants of US mostly developed on MS media. The best result with the IC50 value of the DPPH radical scavenging activity of US in vitro regenerated plants (bulbs) was 2516.50 μg/mL, and the IC50 value of the ABTS radical scavenging activity was 681.43 μg/mL for the Aksay population. The IC50 value of the DPPH radical scavenging activity of US in vitro regenerated plants (leaves) was lower compared to bulbs. The antioxidant activity was not detected for in vitro regenerated plants (bulbs), for the plants introduced to the Botanical Garden from the Aksarsay US_3_B, and for the plants introduced to the Botanical Garden collected in the Beldersay US_4_B.

For *U. victoris,* the study results revealed variations in antioxidant activity even within the UV samples from different populations. The IC50 value for DPPH radical scavenging activity of the UV_2 sample collected from the vicinity of Nilu village in the Surkhandarya region was the highest at 1367.95 μg/mL, with an IC50 value of 533.82 μg/mL for ABTS radical scavenging activity, demonstrating an excellent antioxidant activity of chemical compounds among all UV extracts of the plants collected from nature. Among the UV samples collected from nature and cultivated in the Botanical Garden, the UV_9 sample exhibited the best antioxidant activity, with an IC50 value of 685.70 μg/mL for DPPH radical scavenging activity and 490.03 μg/mL for ABTS radical scavenging activity. The antioxidant activity of UV callus extracts surpassed the IC50 value for the ABTS radical scavenging activity compared to the DPPH radical scavenging activity.

The best result with IC50 value for the DPPH radical scavenging activity of in vitro regenerated plants (bulbs) was 3556.05 μg/mL, and the IC50 value of the ABTS radical scavenging activity was 1081.04 μg/mL. For UV, the IC50 value for the DPPH radical scavenging activity for in vitro regenerated plants (leaves) was detected as 1542.79 μg/mL, and the IC50 value of the ABTS radical scavenging activity of the regenerated plants (leaves) was 823.83 μg/mL.

The results of the experiments confirmed that the tissue cultures of US and UV are characterized by low levels of antioxidant activity based on the IC50 values for the DPPH and ABTS radical scavenging activities. In vitro regenerated plants (leaves and bulbs) were characterized with higher data on the DPPH and ABTS scavenging activities based on IC50 values; thus, we could conclude that this source is a potentially useful raw material for the extraction of biologically active compounds to replace natural resources.

## 4. Discussion

The two *Ungernia* Bunge (Amaryllidaceae J.St.-Hil.) species out of six growing in Central Asia are of big interest to pharmaceutical companies: *U. sewertzowii* (US) and *U. victoris* (UV). Local people collect the leaves of the plants from early April until May, leaving only a few populations to regenerate. The perspectivity of the species to totally disappear from nature is real and could happen in the near future.

Lycorine is used to treat acute and chronic bronchitis; it has strong vomiting and expectorant effects. Lycorine inhibits protein synthesis [11] and weakly inhibits acetylcholinesterase (AChE) and ascorbic acid biosynthesis [13]. The raw materials of US and UV are the main sources of lycorine and galanthamine, although their chemical synthesis has been achieved [37]. With increased demand by pharmaceutical companies and the limited availability of plant sources, the biosynthesis of lycorine and galanthamine by plant in vitro systems is considered an alternative option for the sustainable production of these valuable natural products. The presented protocols for in vitro propagation of US and UV could be the solution for the conservation of these species’ natural populations. The callus and regenerated plants (microbulbs) of US and UV are good and rapidly propagated sources of target compounds.

The result of research on in vitro propagation of two medicinal plants, US and UV, revealed the high potential of the segments of germinated seeds as the source of explant. The different parts of the plants, including the bulb scales, bulb bottom, leaves, seeds, and segments of the germinated seeds (cotyledon, hypocotyl, and radicle), were used as explants. Only segments of the germinated seeds were transformed into well-formed callus and regenerated plants; however, the processes of callusogenesis and direct/indirect organogenesis were observed on the bulb scales of US and UV as well, but no adult plants were developed.

For the first time, the protocol for microclonal propagation of *U. victoris* was developed by Kunakh et al., 2009 [32]. The authors used the bulb scales as explants (Table 5). The sterilized bulb scales were cut into pieces and placed on a nutrient medium Vch with NAA 0.5 mg/L + Kin 0.02 mg/L. The in vitro regenerated UV bulbs were developed in 30–60 days on 30% of the explants. The available analog on microclonal propagation of *U. victoris* developed by Kunakh et al., 2009 [32], UA 85571 C2 (2009) (A01H4/00, C12N 5/04) allowed for more than 1 mln bulbs from one bulb within 1.5–2 years. The developing stage of the regenerated bulbs was appropriate for 5–7-year-old plants. Our protocols allow for 100–150 bulbs of well-developed, two-leaved, and rooted plants from one explant within 6 months after transferring the callus to the appropriate media. The development phase of the regenerated plants was equal to two years—old galophyte species.

The majority of plant specialists raised the problem of the genetic stability of callus and in vitro regenerated plants. Genetic variability in microbulbs of UV was studied by Bublyk et al. (2012) [38] with the use of RAPD markers to assess the genetic stability of in vitro regenerated plants produced through direct regeneration and indirect organogenesis from long-term callus culture. The micropropagation of UV through direct regeneration resulted in high genetic stability of the produced plants and was recommended for use for large-scale propagation of this rare medicinal plant and conservation of its natural resources. Callus tissues, even upon long-term culturing, display a low level of genetic variation in UV plants [38]. The results confirmed the possibility of using US and UV species in conservation programs and as raw materials for the extraction of biologically active compounds.

The alkaloids content, i.e., galanthamine and lycorine, are lower in the callus and in microbulbs in comparison to the intact plants, though alternatives (elicitors, genetic transformation, mutagenesis, etc.) could be used to increase the concentration of the target biologically active compounds [39]. Callus cultures may be used for the induction of variation and selection of lycorine- and galanthamine-rich microbulbs with the use of elicitation, light, or exogenic phytohormones [12,18,39,40,41,42]. With respect to US and UV, we defined that the IC50 values of the DPPH and ABTS radical scavenging activities of the callus of US and UV were lower than those obtained for in vitro regenerated plants. The antioxidant activity features of the plants from natural populations and from the Tashkent Botanical Garden were considerably higher than those of in vitro plants. However, the micropropagation of US and UV could be used as a starting point for further studies in this area, leading to progress in the biotechnological production of these valuable alkaloids from in vitro regenerated plants for use in medicinal purposes and for plant conservation.

## 5. Conclusions

The use of in vitro regenerated plants as the source of biologically active compounds is a perspective for the conservation of the natural populations of *U. sewertzowii* and *U. victoris*. The research results of Bublik et al. (2012) [38] showed that regenerated plants were genetically stable; no clonal variations were detected in the plants developed by indirect organogenesis. The callus and the regenerated plants could serve as the source for the extraction of biologically active compounds; therefore, elicitation, light, or exogenic phytohormones could increase the concentration of target compounds [12,18,39,40,41,42]. The use of developed protocols for *U. sewertzowii* and *U. victoris* could provide regenerated plants to replace the raw material from natural populations or established plantations and, in this way, decrease the pressure on natural populations.

## Figures and Tables

**Table 1:** Sites of collection of seeds and bulbs of *Ungernia sewertzowii* (**A**) and *Ungernia victoris* (**B**).

(**A**). *Ungernia sewertzowii* (three populations)				
Site of collection	Date of collection D/M/Y	Coordinates		
		Longitude	Latitude	Altitude, masl
Western Tien Shan, Great Chimgan mountain, Aksay and Katta Kok Say river banks	8 April 2020	41.512175	70.050850	2434
Western Tien Shan, Pskem range, Aksarsay river, vicinity to Nanay village	25 June 2021	41.690587	70.234465	2832
Tashkent region, Chatkal range, vicinity to Beldersay river	7 June 2021	41.476467	69.975805	2275
Gulkamsay	7 June 2021	41.486467	69.875805	2375
(**B**). *Ungernia victoris* (two populations)				
Site of collection	Date of collection D/M/Y	Coordinates		
		Longitude	Latitude	Altitude, masl
Pamir Alay, Gissar range, Saukbulak mountain, 10 km from Padang village	29 May 2021	38.274326	67.290877	1838
Pamir Alay, Gissar range, basin of Sangardak river, right bank, vicinity of Sangardak village	31 May 2021	38.556908	67.502094	1384

**Table 2:** Callusogenesis on the segments of germinated seeds of *Ungernia sewertzowii* and *Ungernia victoris*.

No.	Segment of Grown Seed	*Ungernia sewertzowii*		*Ungernia victoris*	
		The Number of Explants with a Developed Callus	The Number of Microbulbs on Each Explant	The Number of Explants with a Developed Callus	The Number of Microbulbs on Each Explant
1	Hypocotyl	60–80%	150–200	60–80%	150–200
2	Cotyledon	50–60%	100–120	50–60%	100–120
3	Radicle	10–15%	0	10–15%	0

**Table 3:** *Ungernia sewertzowii*. The induction of callusogenesis and the quantity of microbulbs on nutrient media with different combinations of phytohormones. Sources of explants: hypocotyl and cotyledon.

No.	Combination of Phytohormones	Callusogenesis, %	The Number of Microbulbs on One Explant
1	M5 24D 0.5 mg/L + BAP 0.5 mg/L	40 ± 0.2	100
2	M56 2.4D 0.5 mg/L + Kin 0.5 mg/L	95 ± 0.2	150
3	M87 2.4D 0.5 mg/L + Zea 0.5 mg/L	95 ± 0.2	150
4	M40 IAA 2.0 mg/L + BAP 0.5 mg/L	30 ± 0.16	3
5	M44 IAA 0.5 mg/L + Kin 0.5 mg/L	40 ± 0.1	4–5
6	M68 IAA 0.5 mg/L + Kin 0.5 mg/L	10 ± 0.1	3–5
7	M32 IAA 0.5 mg/L + BAP 0.5 mg/L	10 ± 0.1	4–5
8	M17 IAA 0.5 mg/L + BAP 0.5 mg/L	0	4–5

**Table 4:** Direct and indirect organogenesis of *Ungernia sewertzowii* and indirect organogenesis of *Ungernia victoris* on the nutrient media of Murashige and Skoog, 1962 [28] with various combinations of phytohormones. Sources of explants: hypocotyl and cotyledon.

Nutrient Media	Number of Microbulbs Per Explant in Direct Organogenesis	Number of Microbulbs Per Explant in Indirect Organogenesis
M44 NAA 0.5 mg/L + Kin 0.5 mg/L	4–5	100–150
M68 IAA 0.5 mg/L + Kin 0.5 mg/L	3–5	100–150
M32 NAA 0.5 mg/L + BAP 0.5 mg/L	4–5	100–150
M17 IAA 0.5 mg/L + BAP 0.5 mg/L	4–5	100–150

**Table 5:** The difference of the research results with analog protocol.

#		*Ungernii sewertzowii*	*Ungernia victoris*	Patent UA 85571 C2 (Ukraine)
1	The source of explant	Segments of germinated seeds: cotyledon, hypocotyl, and radicle	Segments of germinated seeds: cotyledon, hypocotyl, and radicle	Scales of bulbs
		Advantages: low degree of contamination, high degree of regeneration, year-round access to material	Advantages: low degree of contamination, high degree of regeneration, year-round access to material	Disadvantages: high degree of contamination, material availability only in spring and summer
2	Nutrient media	Nutrient media by Murashige and Skoog (1962) [28]	Nutrient media by Murashige and Skoog (1962) [28]	Nutrient media by Vollosovich (1979) [35]
3	Phytohormone content	Inducing in vitro culture: A. 2.4D 0.5 mg/L + BAP 0.5 mg/L B. 2.4D 0.5 mg/L +Kin 0.5 mg/L C. 2.4D 0.5 mg/L + Zea 0.5 mg/L Organogenesis (direct/indirect): M17 IAA 0.5 mg/L + BAP 0.5 mg/L M32 NAA 0.5 mg/L + BAP 0.5 mg/L M44 NAA 0.5 mg/L + Kin 0.5 mg/L, and M68 IAA 0.5 mg/L + Kin 0.5 mg/L	Inducing in vitro culture: 2,4D 0.5 mg/L + Kin 0.5 mg/L No direct organogenesis observed The indirect organogenesis were induced on MS: M17 IAA 0.5 mg/L + BAP 0.5 mg/L M32 NAA 0.5 mg/L + BAP 0.5 mg/L M44 NAA 0.5 mg/L + Kin 0.5 mg/L, and M68 IAA 0.5 mg/L + Kin 0.5 mg/L	Inducing in vitro culture: A. IAA 1.8–2.2 mg/L + Kin 0.8–1.2 mg/L Caseine hydrolysate 450–550 mg/L Mesoinosite 70–100 mg/L B. IAA 0.5 mg/L + Kin 0.02 mg/L Caseine hydrolysate 50 mg/L Mesoinosite 20 mg/L C. IAA 2.0 mg/L + Kin 0.02 mg/L Caseine hydrolysate 50 mg/L Mesoinosite 20 mg/L
4		Proliferation: The same media and phytohormones concentrations were used	Proleferation: The same media and phytohormones concentrations were used	Proliferation: IAA 2.0 mg/L + Kin 0.02 mg/L Caseine hydrolysate 40–60 mg/L Mesoinosite 10–30 mg/L
5		Proliferation: Callusogenesis 2.4D 0.5 mg/L + Kin 0.5 mg/L Organogenesis: M17 IAA 0.5 mg/L + BAP 0.5 mg/L M32 NAA 0.5 mg/L + BAP 0.5 mg/L, and M68 IAA 0.5 mg/L + Kin 0.5 mg/L	-	Proliferation: IAA 0.2–0.5 mg/L + Kin 0.02–0.1 mg/L Caseine hydrolysate 40–60 mg/L Mesoinosite 15–25 mg/L
6		Stimulation of rhizogenesis: NAA 2.5 mg/L + BAP 0.5 mg/L + TDZ 0.3 mg/L	Stimulation of rhizogenesis: NAA 2.5 mg/L + BAP 0.5 mg/L + TDZ 0.3 mg/L	
7		Stimulation of rhizogenesis: NAA 0.5 mg/L	Stimulation of rhizogenesis: NAA 0.5 mg/L	

## Data Availability

The original contributions presented in the study are included in the article/Supplementary Material, further inquiries can be directed to the corresponding authors.

## Supplementary Materials

The following supporting information can be downloaded at: https://www.mdpi.com/article/10.3390/plants13141966/s1.

## Abbreviations

2.4D	2,4-Dichlorophenoxyacetic acid
ABTS	2,2-Azinobis (3-ethylbenzothiazoline)-6-sulphonic acid
atm.	Atmosphere, air pressure measurement at sea level at a temperature of 15 °C.
B5	Nutrient media by Gamborg et al. (1968) [30]
BAC	Biologically active compounds
BAP	6-Benzylaminopurine
DPPH	2,2-Diphenyl-1-picrylhydryl
DW	Dry weight
IAA	Indole-3-acetic acid
IC50	Half-maximal inhibitory concentration
Kin	Kinetin
l	Liter
M	Shortage from MS
masl	Meters above sea level
mg	Milligram
mM	Millimol
MS	Murashige and Skoog, nutrient media by Murashige and Skoog (1962) [28]
N6	Nutrient media by Chu et al. (1975) [29]
NAA	1-Naphthaleneacetic acid
nm	Nanomol
US	*Ungernia sewertzowii*
UV	*Ungernia victoris*
Vch	Vollosovich, nutrient media by Vollosovich (1979) [35]
WPM	Woody plant medium, nutrient media by Lloyd G. and McCown (1980) [31]
Terminology	
Rhizogenesis	the development of the roots.
Gemmogenesis	the development of the stems.
Gemmorhizogenesis	the development of stems and roots.
Callusogenesis	the formation of calli in damaged plant tissue.
Direct organogenesis	the production of direct buds or shoots from tissue with no intervening callus stage.
Indirect organogenesis	the process by which plant organs are derived from a calli mass in the explant.
