# Supplementary material for: Conservation Potential Trough In Vitro Regeneration of Two Threatened Medicinal Plants Ungernia sewertzowii and U. victoris
Source: Plants (Basel). 2024 Jul 18;13(14):1966. doi: 10.3390/plants13141966 (PMC11280894; doi:10.3390/plants13141966)
Supplement: Supplementary file 1 [file plants-13-01966-s001.zip › plants-3043142-supplementary.pdf]

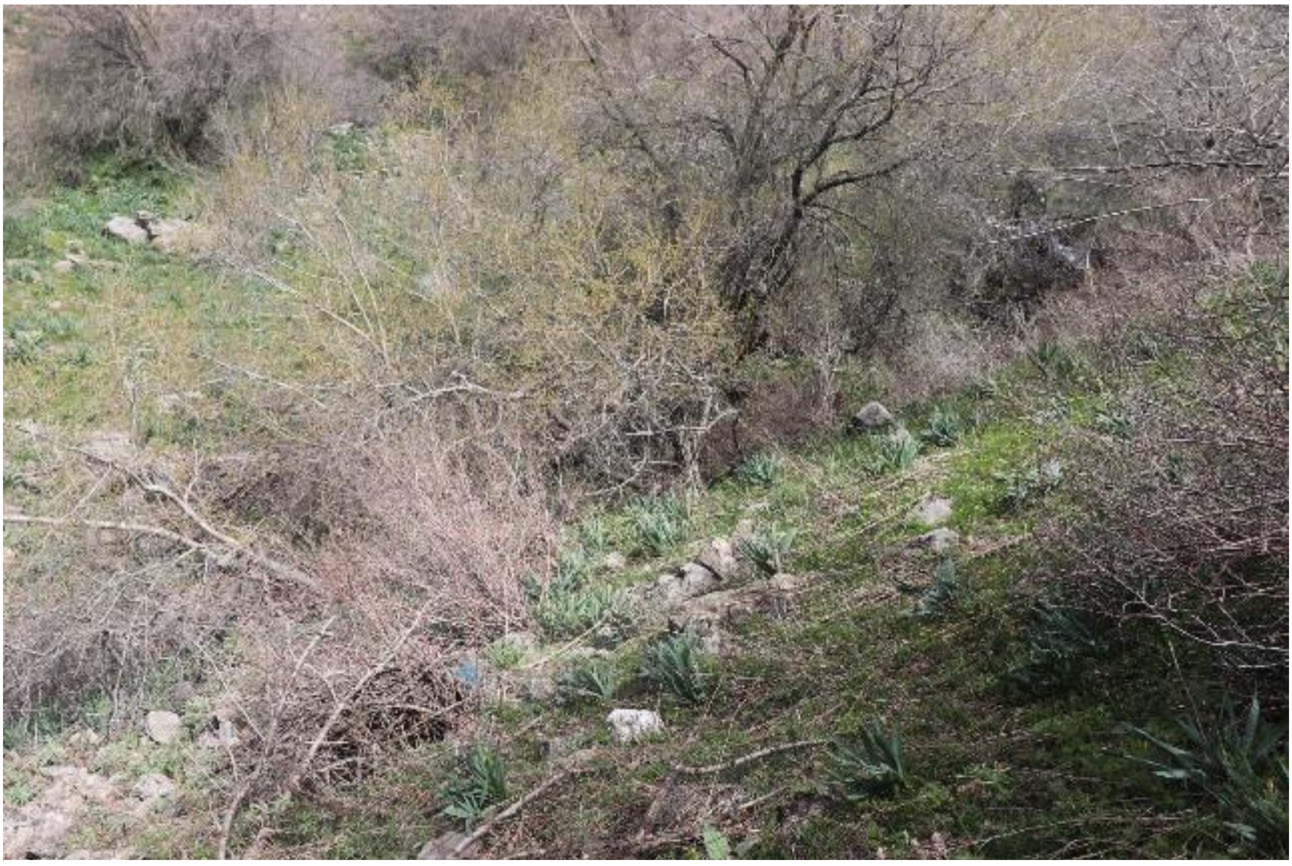

Figure S1. *Ungernia sewertzowii*. Natural populations distributed on the stony slopes of the Pskem range, Western Tien-Shan, the Aksarsay River, in the vicinity of Nanay village. 06.05.2022. Photo by Mustafina F.U.

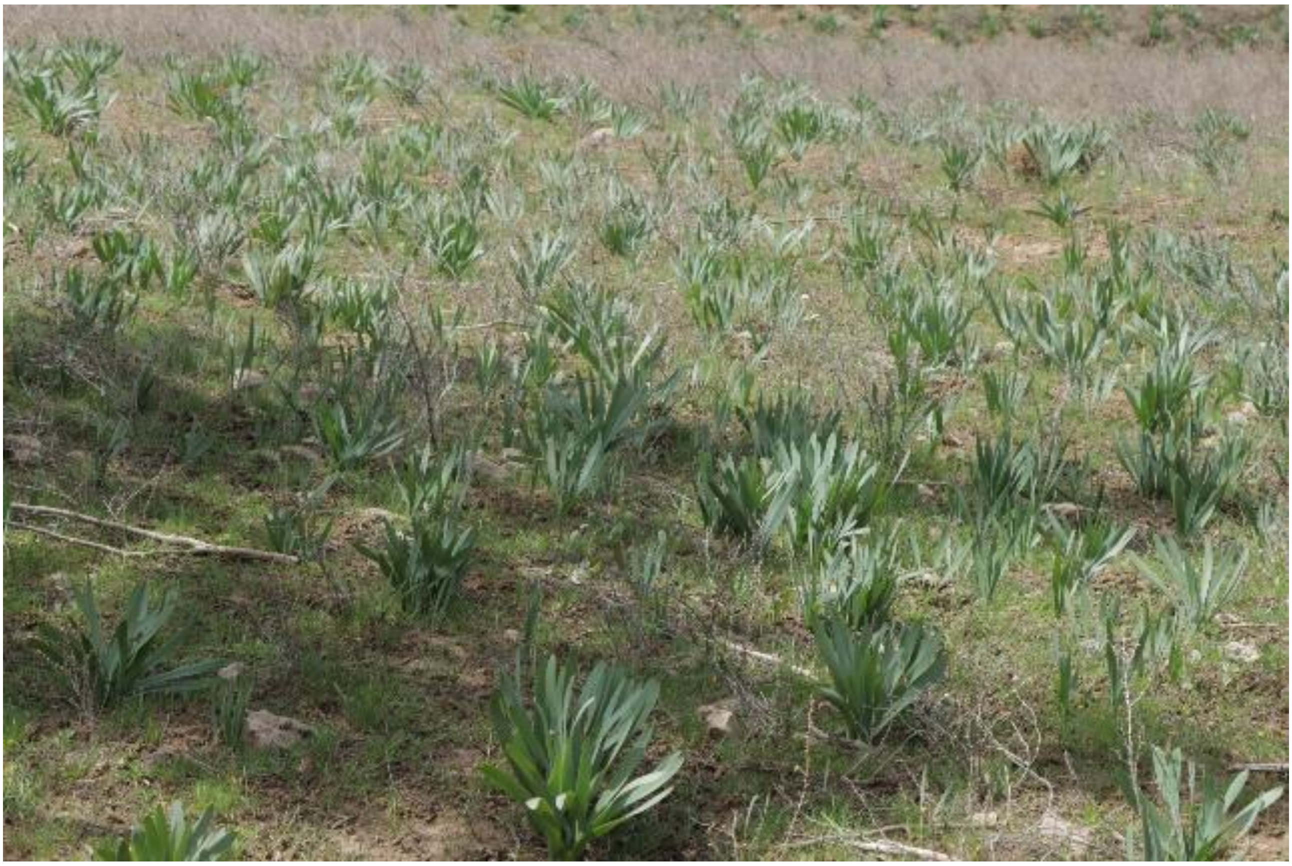

Figure S2. *Ungernia victoris*. Natural populations distributed on the slopes of the Gissar range, Pamir Alay, basin of the Sangardak river, right bank, in the vicinity of Sangardak village. 28.03.2022. Photo by Turdiev D.T.

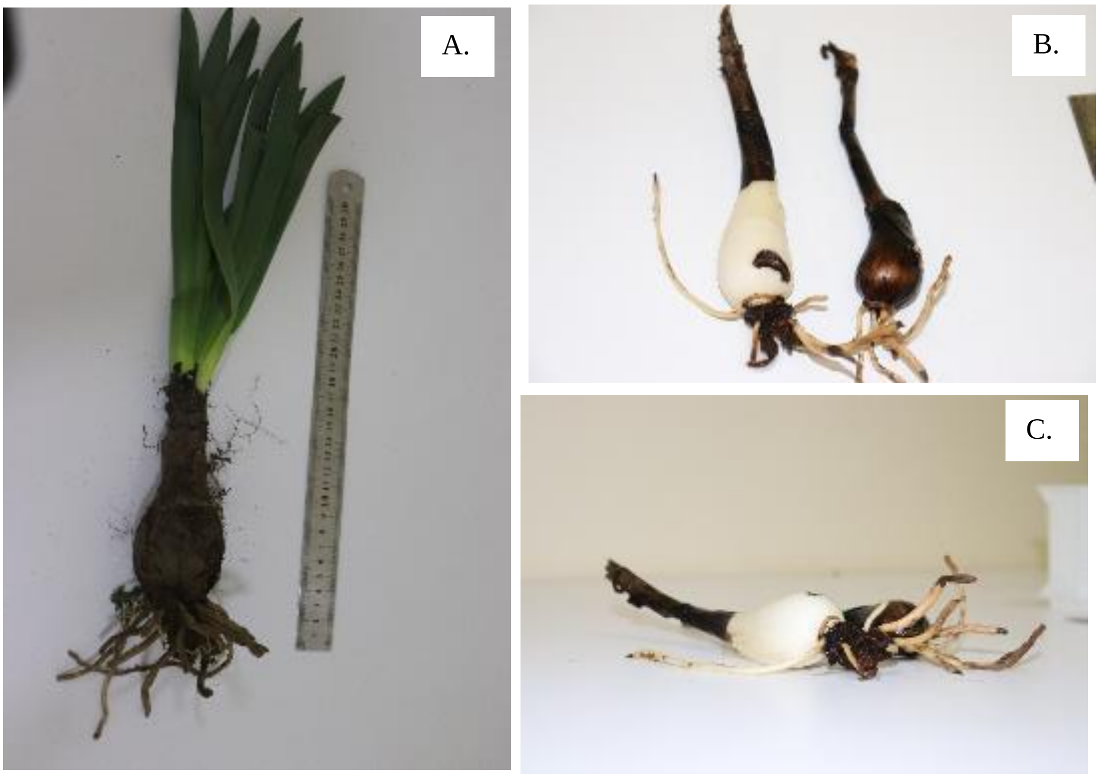

Figure S3. *Ungernia sewertzowii*. Bulbs. A. Collected in the Tashkent region, Chatkal range, vicinity of the Beldersay river. 06.05.2022. B. Plants introduced in the Tashkent Botanical Garden. 07.07.2022. The bulbs of the plants from natural populations and from the Botanical Garden did not differ in size, color, consistency. Photos by Mustafina F.U.

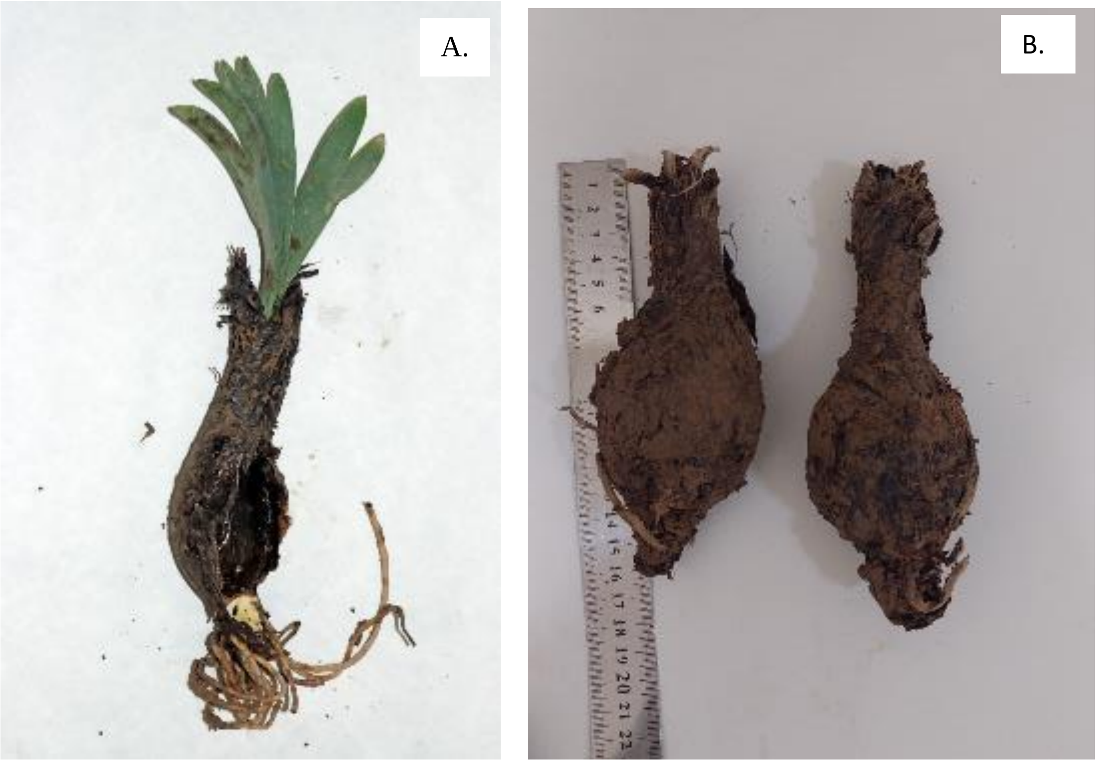

Figure S4. *Ungernia victoris*. Bulbs. A. Collected in Pamir Alay, Gissar range, basin of the Sangardak river, right bank, in the vicinity of Sangardak village. 28.03.2022. B. Plants Introduced in the Tashkent Botanical Garden. 23.11.2022. The bulbs of the plants collected from natural populations and the bulbs of the plants introduced in the Botanical Garden did not differ in size, color, and consistency. Photos by Mustafina F.U.

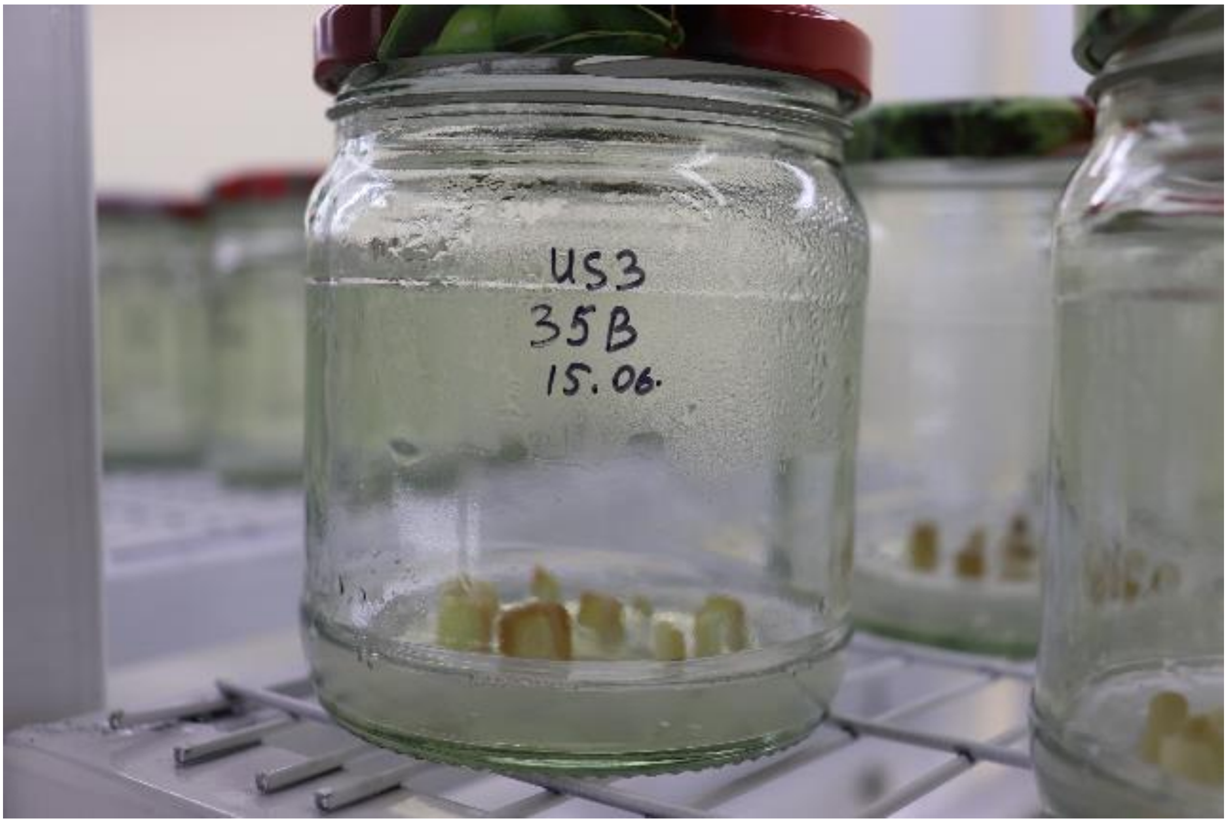

Figure S5. *Ungernia sewertzowii*. Using bulb scales as the explants for *in vitro* propagation of *Ungernia* species. The nutrient medium is by Murashige and Skoog (1962). Callusogenesis, indirect organogenesis, and direct/indirect somatic embryogenesis on Murashige and Skoog (1962) and Vollosovich (1979) were induced on bulb scales when tested up to 183 phytohormone combinations, but none of the adult plants resulted from observed processes. Callus induction was observed on the segments of bulb scales of *U. sewertzowii* and *U. victoris* on Murashige and Skoog (1962) nutrient media, and somatic embryos were determined on Vollosovich (1979) nutrient media. Foto by Mustafina F.U.

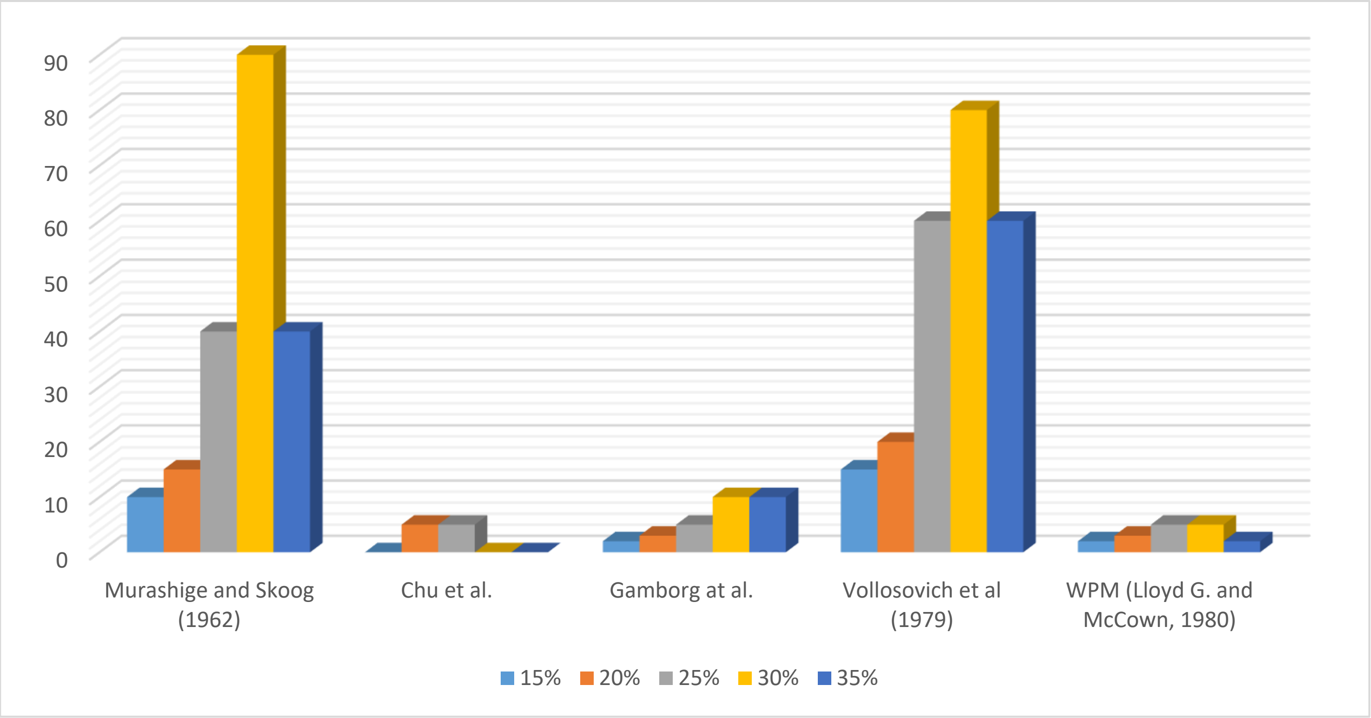

Figure S6. Structural changes on explants (%) on different nutrient media and sucrose concentrations for *in vitro* propagation of *U. sewertzowii* and *U. victoris* with the scales of the bulbs as the source of explants. Almost 90% of the explants showed changes in their structure on Murashige and Skoog (1962) media with a sucrose concentration 30%.

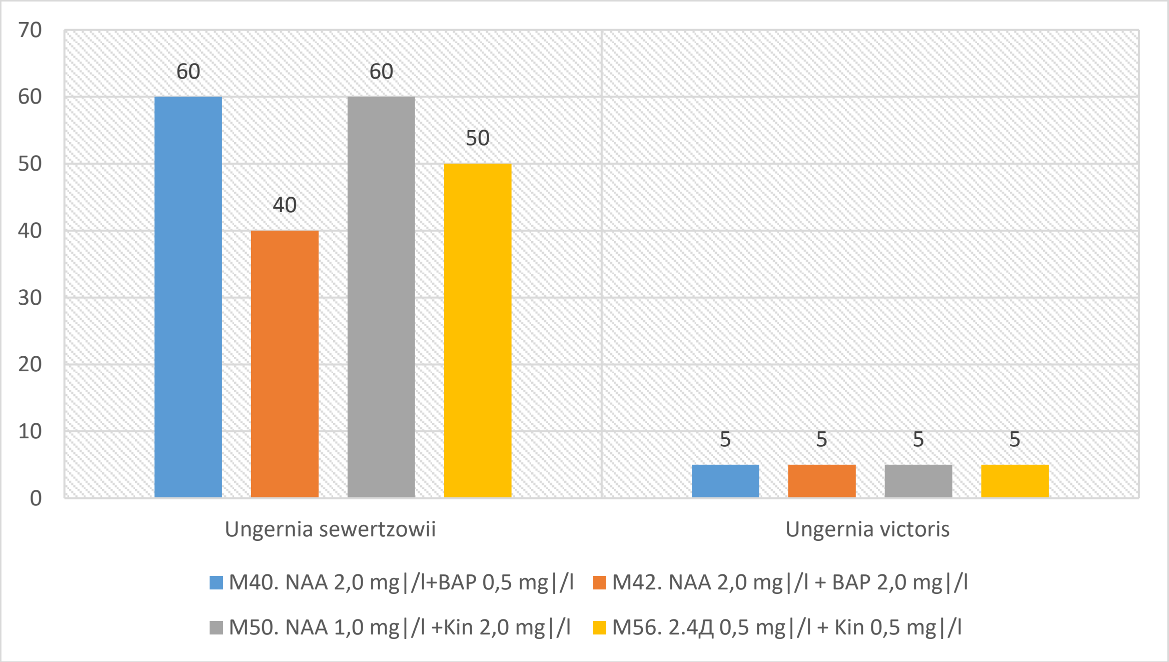

Figure S7. *Ungernia sewertzowii* and *U. victoris*. The share of explants (%) with direct somatic embryogenesis on the nutrient media by Murashige and Skoog (1962). Source of explants: bulb scales. Direct somatic embryogenesis on the bulb scales of US on MS with phytohormones M40 NAA 2.0 mg/l + BAP 0.5 mg/l and M56 2.4D 0.5 mg/l + Kin 0.5 mg/l. No somatic embryogenesis was observed for *U. victoris* on Murashige and Skoog (1962) media.

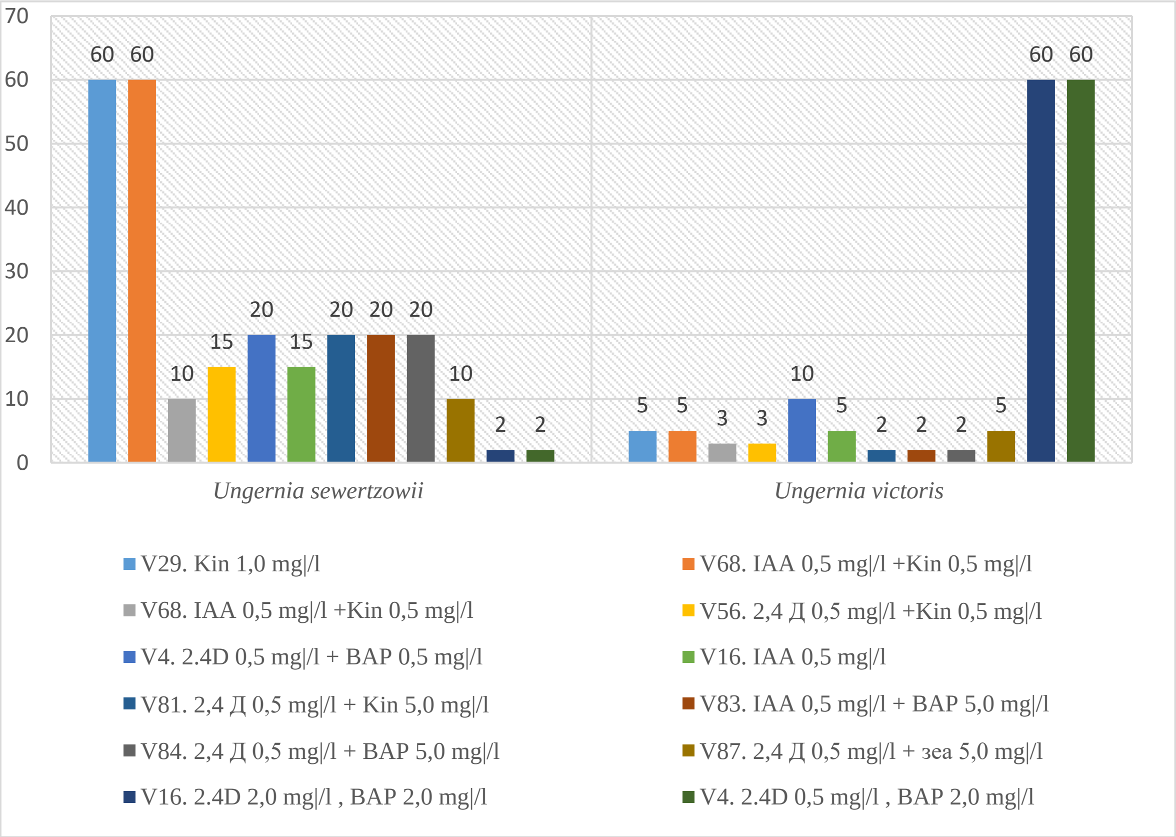

Figure S8. *Ungernia sewertzowii* and *Ungernia victoris*. Direct somatic embryogenesis on nutrient media by Vollosovich et al. (1979), %. Source of explants: bulb scales. Direct somatic embryogenesis on the bulb scales of *U. sewertzowii* on Vollosovich et al. (1979) with phytohormones V29 Kin 1.0 mg/l, V68 IAA 0.5 mg/l +Kin 0.5 mg/l, V56 2.4D 0.5 mg/l +Kin 0.5 mg/l, V4 2.4D 0.5 mg/l, V16 IAA 0.5 mg/l, V81 2.4D 0.5 mg/l + Kin 5.0 mg/l, V83 IAA 0.5 mg/l + BAP 5.0 mg/l, V84 2.4D 0.5 mg/l + BAP 5.0 mg/l, and V87 2,4D 0.5 mg/l + Zea 0.5 mg/l. Direct somatic embryogenesis on the bulb scales of *U. victoris* with phytohormones V16 IAA 0.5 mg/l, and V4 2.4D 0.5 mg/l.

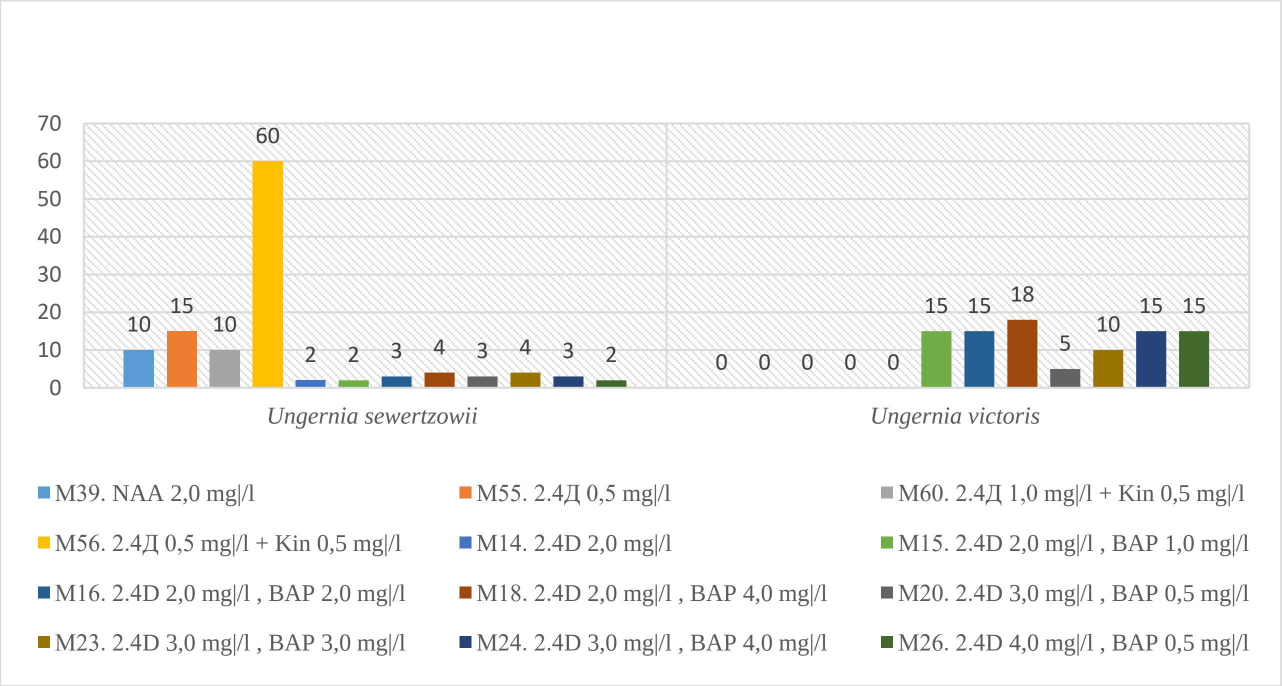

Figure S9. *Ungernia sewertzowii* and *U. victoris*. The share of explants (%) with callusogenesis on the nutrient medium by Murashige and Skoog (1962). Source of explants: bulb scales. Callusogenesis on the bulb scales of US on nutrient medium by Murashige and Skoog (1962) with phytohormones M39 NAA 2.0 mg/l, M55 2.4D 0.5 mg/l, M60 2.4D 1.0 mg/l + Kin 0.5 mg/l, M56 2.4D 0.5 mg/l + Kin 0.5 mg/l, and week callus formation or its absence for UV with phytohormone 2.4D 2.0 mg/l, and combination of phytohormones 2.4D 2.0-4.0 mg/l + BAP 0.5-4.0 mg/l.

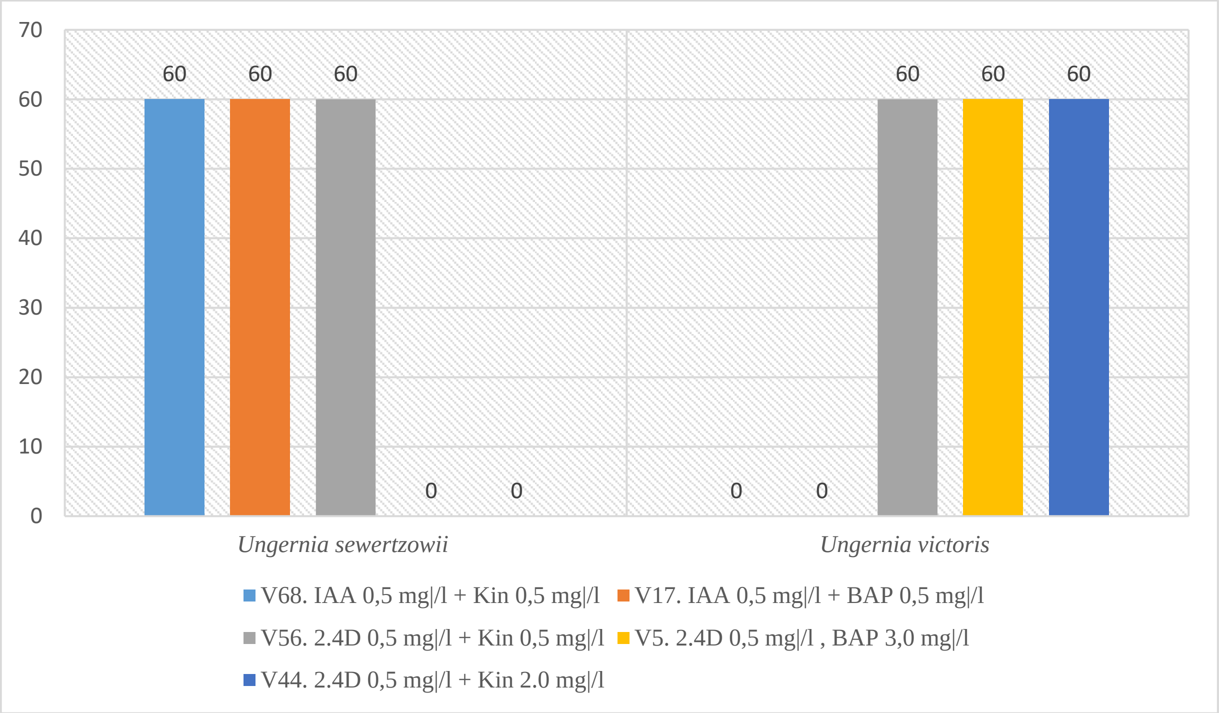

Figure S10. *Ungernia sewertzowii* and *U. victoris*. The share of explants (%) with callusogenesis on nutrient medium by Vollosovich (1979). Source of explants: bulb scales. Callusogenesis was observed on the bulb scales of *U. sewertzowii* on nutrient media by Vollosovich et al. (1979) with the phytohormones V68 IAA 0.5 mg/l + Kin 0.5 mg/l, V17 IAA 0.5 mg/l + BAP 0.5 mg/l, V56 2.4D 0.5 mg/l + Kin 0.5 mg/l. 2. Callusogenesis was observed on the bulb scales of *U.victoris* on nutrient media by Vollosovich et al. (1979) with phytohormones V56 2.4D 0.5 mg/l + Kin 0.5 mg/l, V5 2.4D 0.5 mg/l + BAP 0.5 mg/l, V44 NAA 0.5 mg/l + Kin 0.5 mg/l.

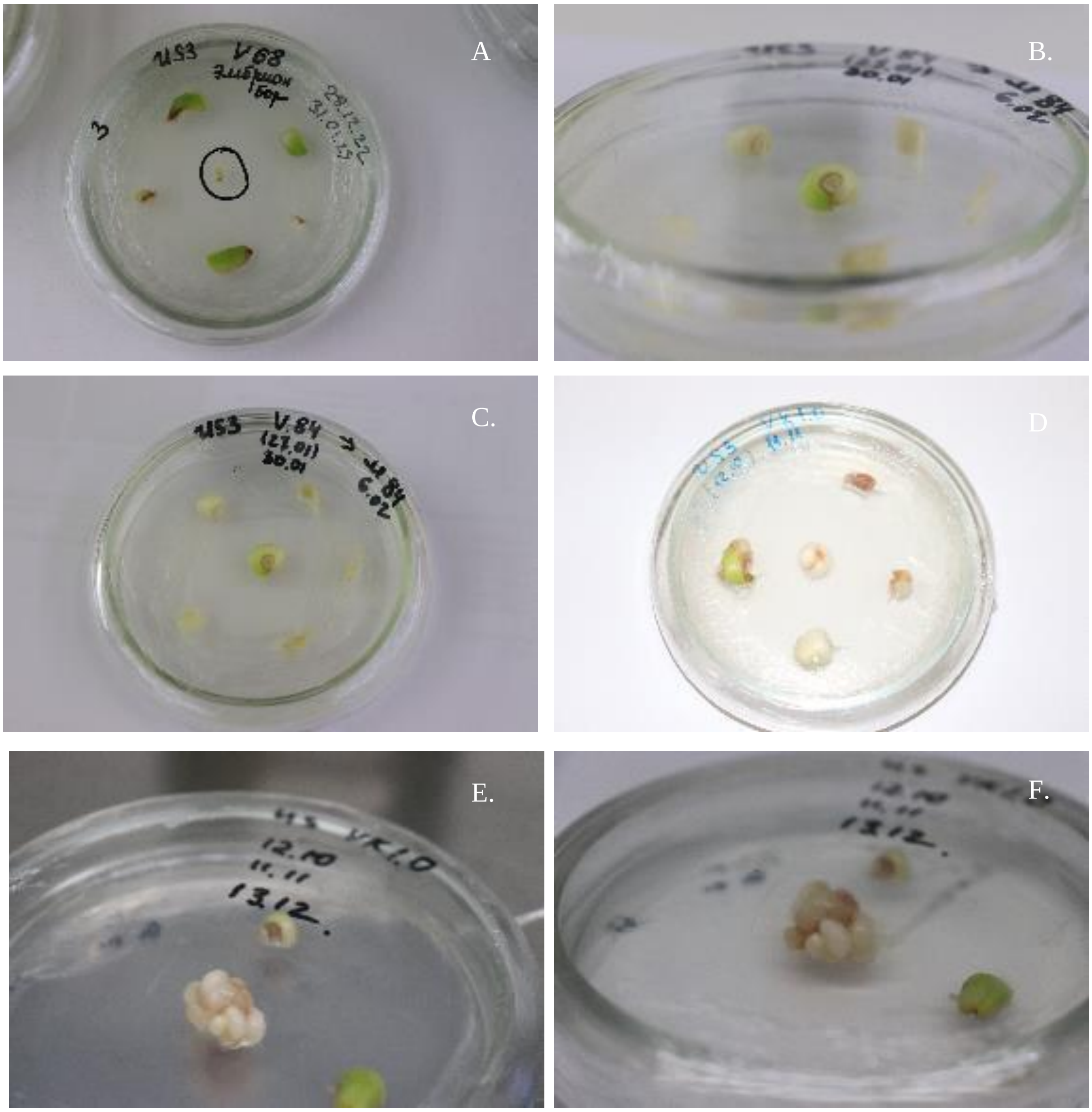

Figure S11. *Ungernia sewertzowii*. Direct somatic embryogenesis of *U. sewertzowii* on nutrient media by Vollosovich (1979). Source of explants: bulb scales. A. V68 IAA 0.5 mg/l + Kin 0.5 mg/l; B. C. V84 2.4D 0.5 mg/l + BAP 5.0 mg/l; D. E. F. VK 1.0 Kin 1.0 mg/l. Indirect somatic embryogenesis was not observed for *U. sewertzowii* on nutrient media by Vollosovich (1979).

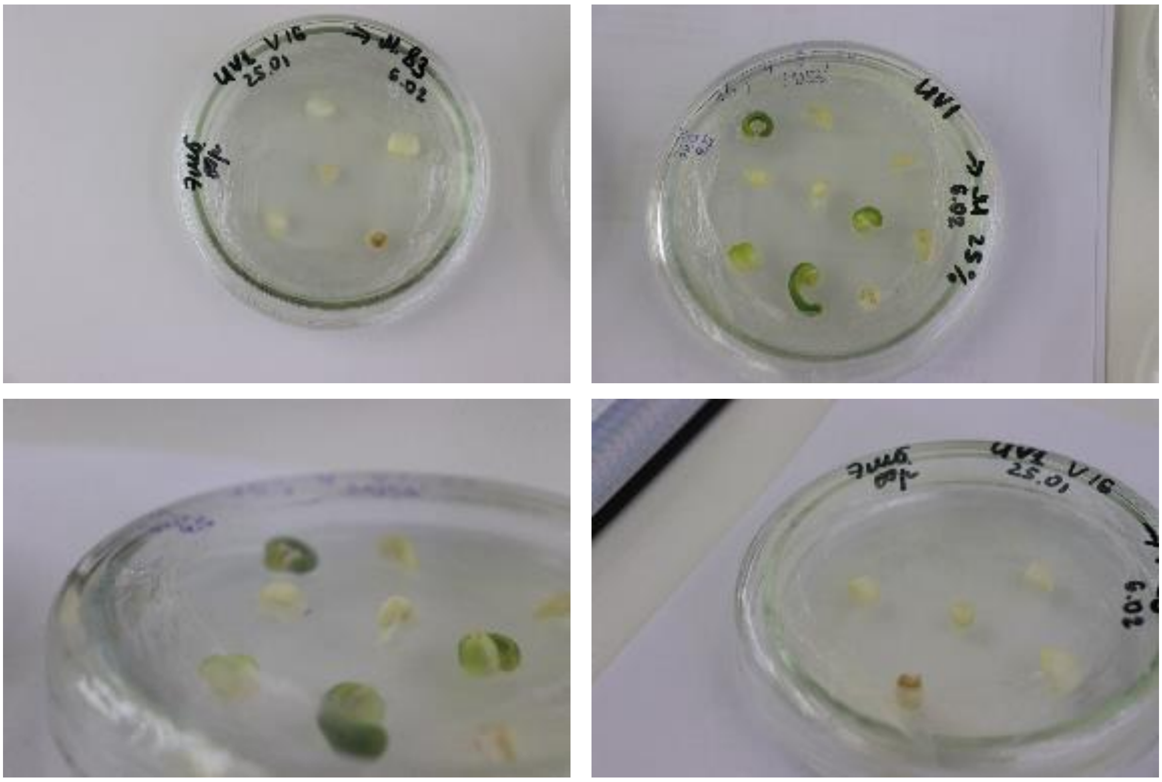

Figure S12. *Ungernia victoris*. Direct somatic embryogenesis of *U. victoris* on nutrient media by Vollosovich (1979). Source of explants: bulb scales. V16 2.4D 2.0 mg/l + BAP 2.0 mg/l.

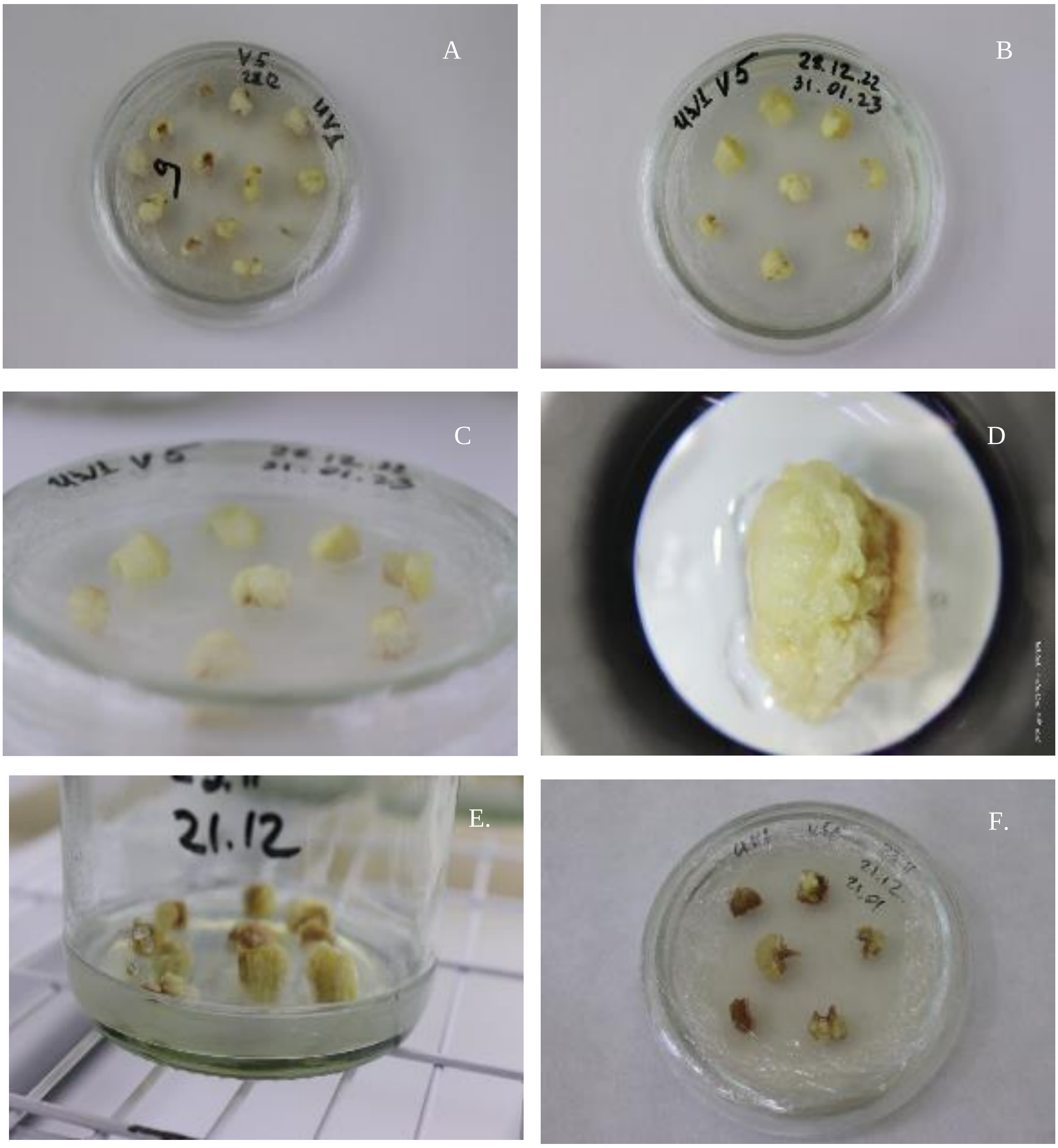

Figure S13. *Ungernia victoris.* Callusogenesis on nutrient media by Vollosovich (1979). Source of explants: bulb scales. A, B, C. V5 2.4D 0.5 mg/l + BAP 3.0 mg/l. D, E, F. V56 2.4D 0.5 mg/l + Kin 0.5 mg/l.

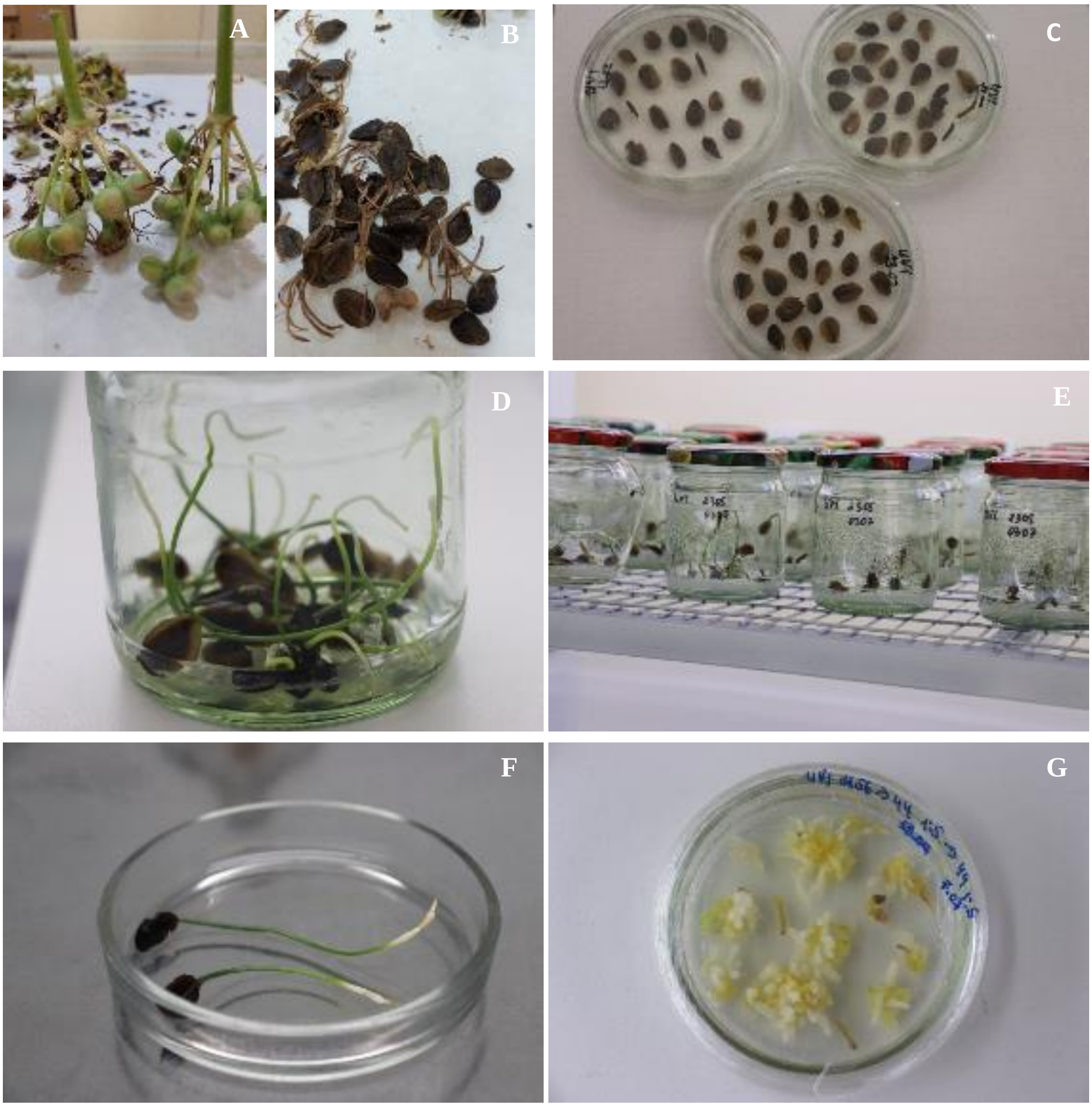

Figure S14. Seed stratification of *Ungernia sewertzowii* and *U. victoris*. A. B. Seeds. C Seeds placed on 25% nutrient media by Murashige and Skoog (1962), in the refrigerator at +5°C. D. E. Seeds placed on 50% nutrient media by Murashige and Skoog (1962), on shelves in a cultural room at +24°C. F. Using germinated seeds as the source of the explant. G. Callusogenesis.

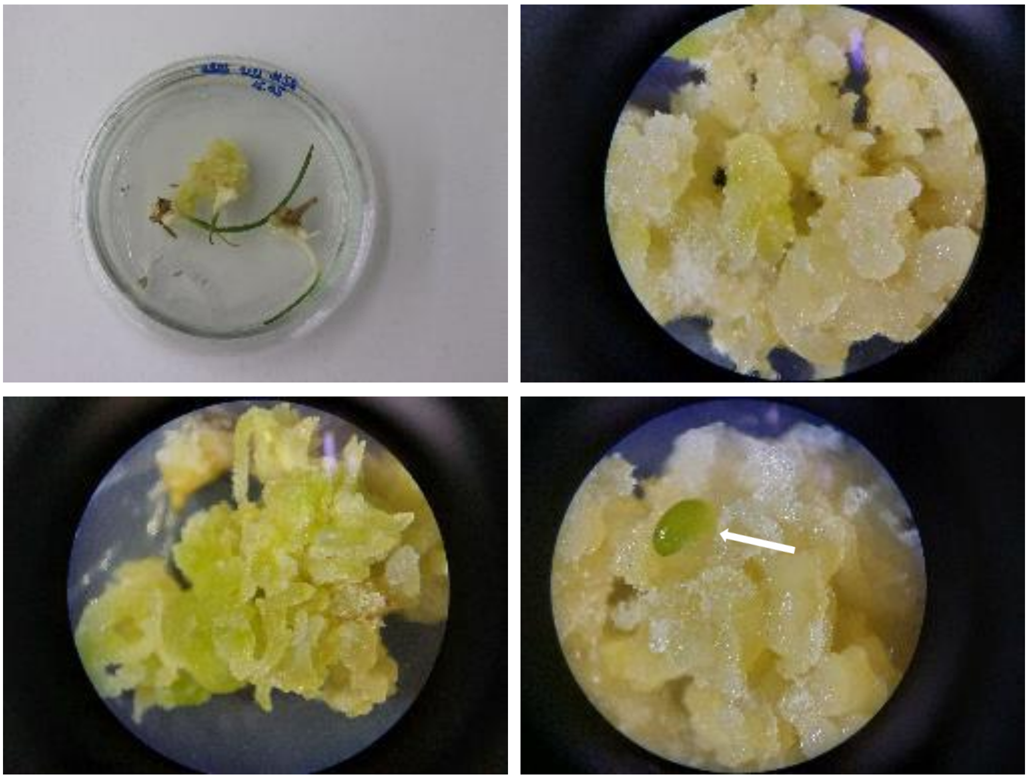

Figure S15. *Ungernia sewertzowii*. Callusogenesis. Nutrient media by Murashige and Skoog (1962). M56 2.4D 0.5 mg/l + Kin 0.5 mg/l. Source of explants: segments of the germinated seeds (hypocotyl, cotyledon, radicle). Rudiments of green buds are shown with an arrow.

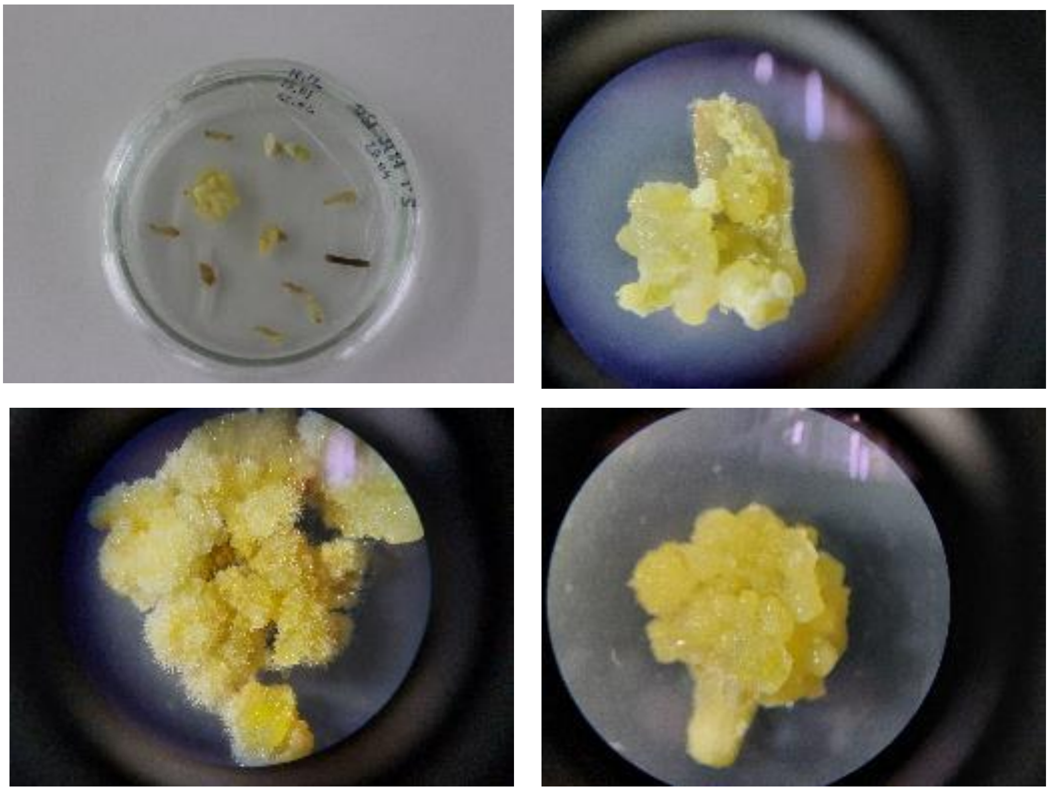

Figure S16. *Ungernia sewertzowii*. Callusogenesis. Nutrient media by Murashige and Skoog (1962). M87 2.4D 0.5 mg/l + Zea 0.5 mg/l. Source of explants: segments of the germinated seeds (hypocotyl, cotyledon, and radicle).

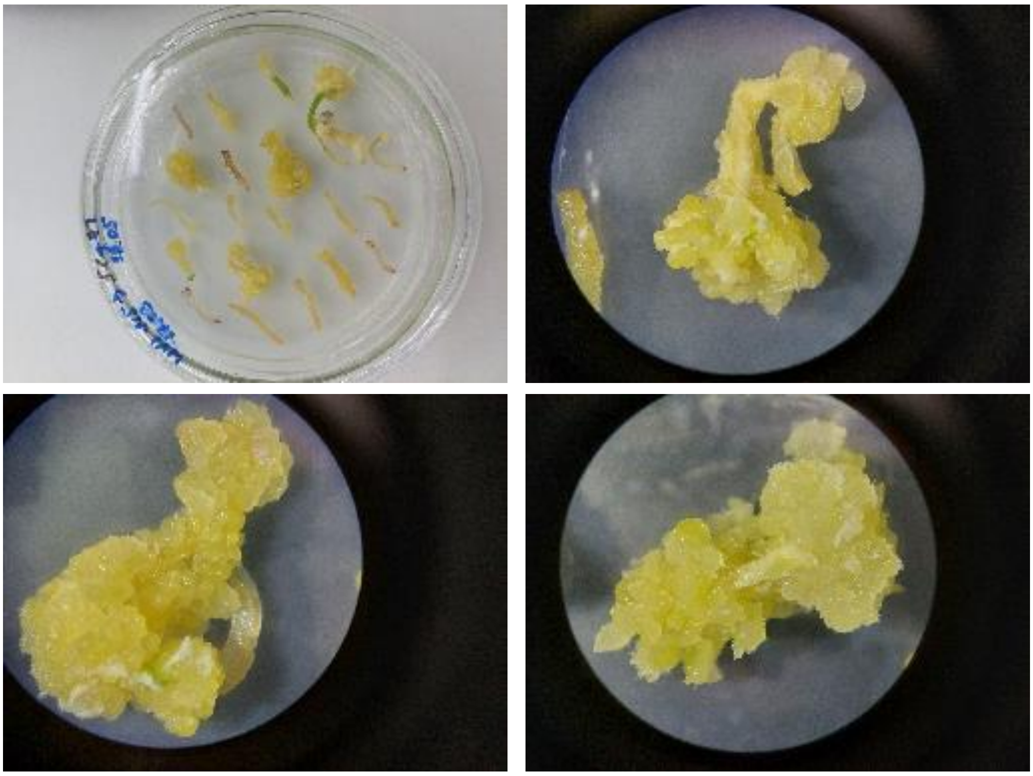

Figure S17. *Ungernia victoris*. Callusogenesis. Nutrient media by Murashige and Skoog (1962). M56 2.4D 0.5 mg/l +Kin 0.5 mg/l. Source of explants: segments of the germinated seeds (hypocotyl, cotyledon, and radicle).

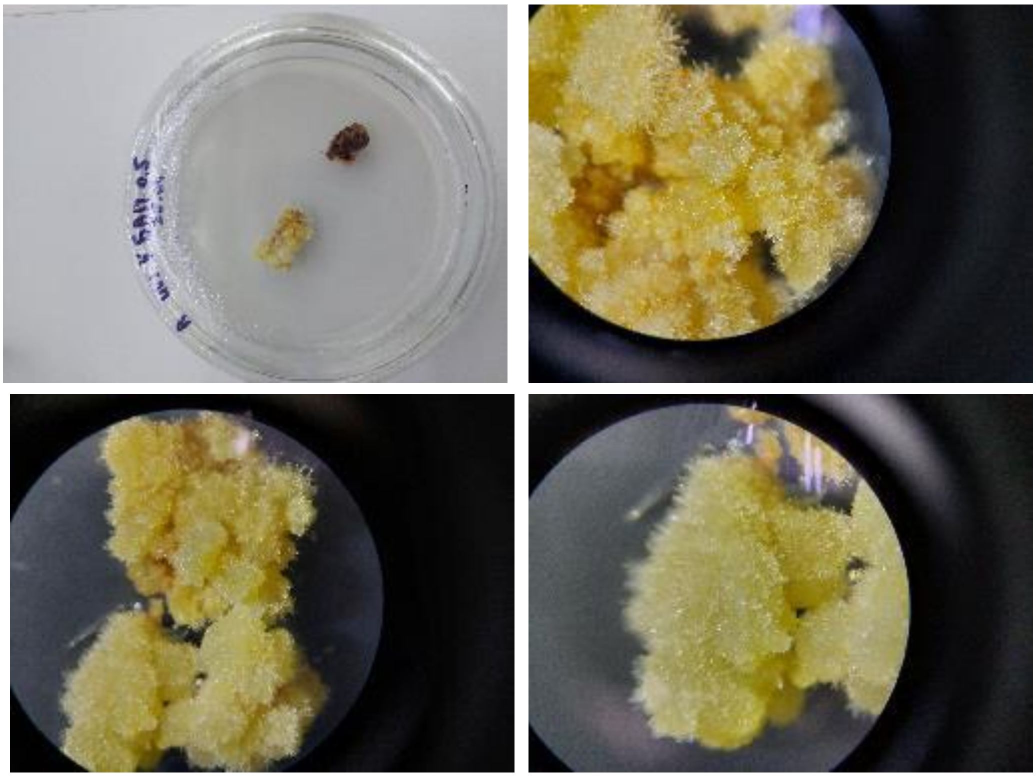

Figure S18. *Ungernia victoris*. Callusogenesis. Nutrient media by Murashige and Skoog (1962). M162 BAP 0.5 mg/l. Source of explants: segments of the germinated seeds (hypocotyl, cotyledon, and radicle).

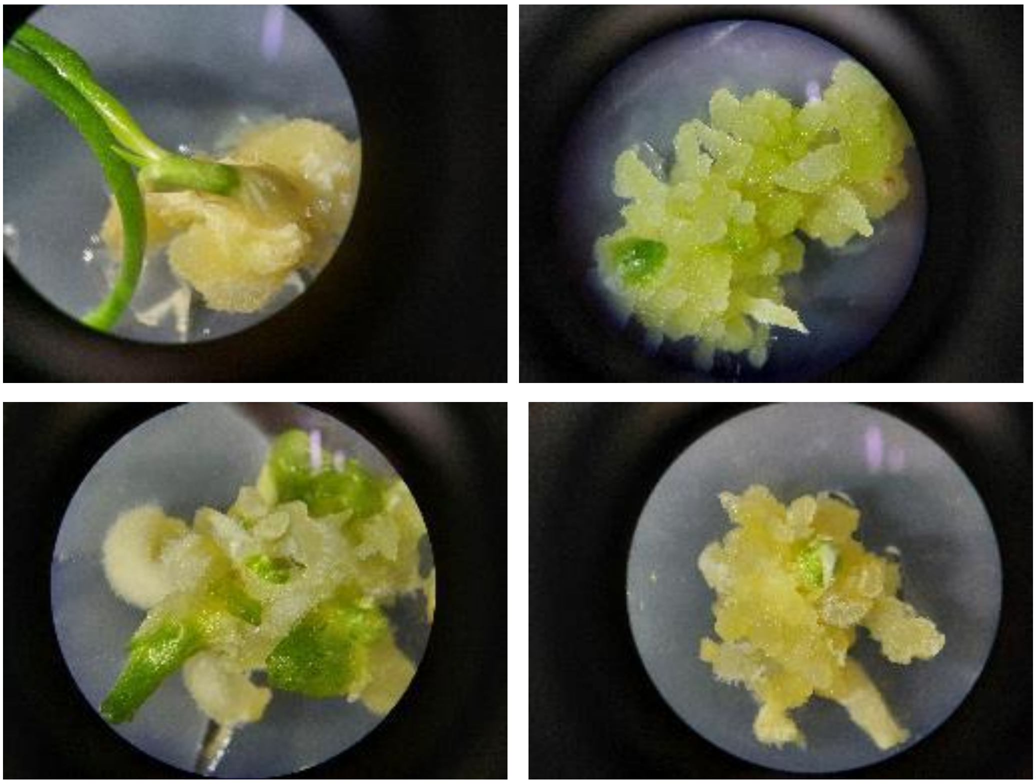

Figure S19. *Ungernia victoris.* Indirect organogenesis on nutrient media by Murashige and Skoog (1962). M17 IAA 0.5 mg/l + BAP 0.5 mg/l. Source of explants: segments of the germinated seeds (hypocotyl, cotyledon, and radicle).

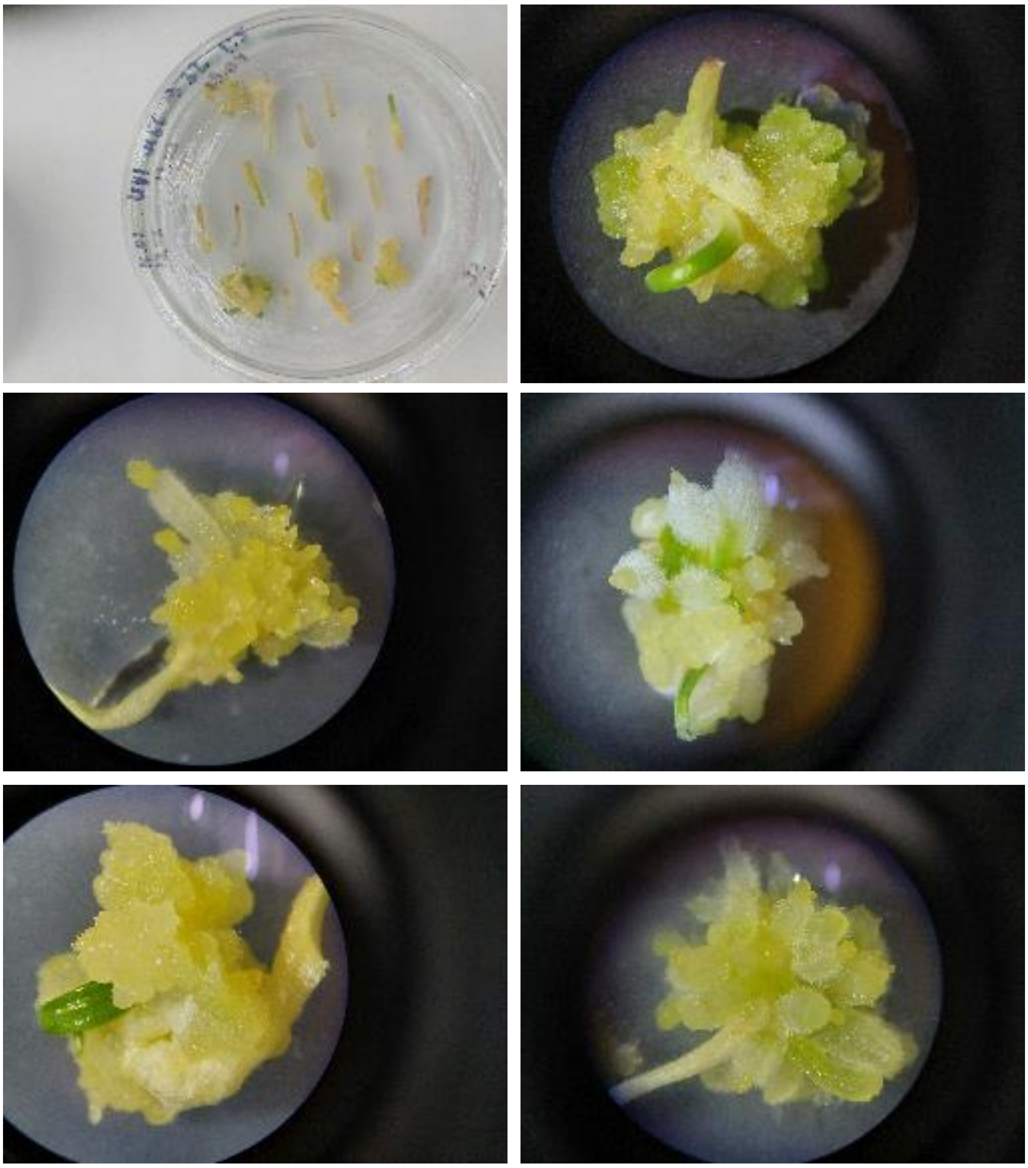

Figure S20. *Ungernia victoris.* Indirect organogenesis on nutrient media by Murashige and Skoog (1962). M32 IAA 0.5 mg/l + BAP 0.5 mg/l. Source of explants: segments of the germinated seeds (hypocotyl, cotyledon, and radicle).

Table S1. Sites of collection of seeds and bulbs of *Ungernia sewertzowii* and *Ungernia victoris*

1. *Ungernia sewertzowii* (3 populations)

	Code of population	Field code	Place of collection	Date of collection	Coordinates		
					Longitude	Latitude	Altitude, masl
pop 1	US1	US(1)-1	Western Tien Shan, Great Chimgan mountain, Aksay and Katta Kok say river.	8/4/2020	41.512175	70.050850	2,434
		US(1)-2		8/4/2020	41.512175	70.050850	2,434
		US(1)-3		8/4/2020	41.512175	70.050850	2,434
		US(1)-4		8/4/2020	41.512175	70.050850	2,434
pop 2	US2	US(2)-5	Western Tien Shan, Pskem range, Aksarsay river, vicinity of Nanay village.	6/25/2021	41.690587	70.234465	2,832
		US(2)-6		6/25/2021	41.690587	70.234465	2,832
		US(2)-7		6/25/2021	41.690587	70.234465	2,832
Pop 3	US3	US3	Tashkent region, Chatkal range, vicinity of Beldersay station.	6/07/2021	41.476467	69.975805	2,275
Pop 4	US4	US4	Gulkamsay	6/07/2021			

2. *Ungernia victoris* (2 populations)

	Code of population.	Field code	Place of collection	Date of collection	Coordinates		
					Longitude	Latitude	Altitude, masl
pop 1	UV1	UV(1)-1	Pamir Alay, Gissar range, Saukbulak mountain, 10 km from Padang village	5/29/2021	38.274326	67.290877	1,838
		UV(1)-2		5/29/2021	38.274326	67.290877	1,838
		UV(1)-3		5/29/2021	38.274326	67.290877	1,838
		UV(1)-4		5/29/2021	38.274326	67.290877	1,838
		UV(1)-5		5/29/2021	38.274326	67.290877	1,838
		UV(1)-6		5/29/2021	38.274326	67.290877	1,838
		UV(1)-7		5/29/2021	38.274326	67.290877	1,838
		UV(1)-8		5/29/2021	38.274326	67.290877	1,838
		UV(1)-9		5/29/2021	38.274326	67.290877	1,838
pop 2	UV2	UV(2)-10	Pamir Alay, Gissar range, basin of the Sangardak river, right bank, vicinity of Sangardak village.	5/31/2021	38.556908	67.502094	1,384
		UV(2)-11		5/31/2021	38.556908	67.502094	1,384
		UV(2)-12		5/31/2021	38.556908	67.502094	1,384
		UV(2)-13		5/31/2021	38.556908	67.502094	1,384
		UV(2)-14		5/31/2021	38.556908	67.502094	1,384

Table S2 – Callusogenesis of of *Ungernia sewertzowii*. Source of explants: bulb scales.

№	Nutrient media by Murashige and Skoog (1962) Combination of phytohormones.	Description of the results: callusogenesis
1.	M39 IAA 2.0 mg/l	The formation of dense yellow calluses is observed, mainly on the basal part of the explants.
2.	M55 2.4D 0.5 mg/l	
3.	M60 2.4D 1.0 mg/l + Kin 0.5 mg/l	
4.	M56 2.4D 0.5 mg/l + Kin 0.5 mg/l	

№	Nutrient madia by Vollosovich (1979). Combination of phytohormones.	Description of the results: callusogenesis
1.	V68 IAA 0.5 mg/l + Kin 0.5 mg/l	The callus formation is weak but observed, ranging from light yellow to dark yellow color.
2.	V17 IAA 0.5 mg/l + BAP 0.5 mg/l	
3.	V56 2.4D 0.5 mg/l + Kin 0.5 mg/l	

Table S3 – Direct/indirect somatic embryogenesis of *Ungernia sewertzowii*. Source of explants: bulb scales.

№	Nutrient media by Murashige and Skoog (1962) Combination of phytohormones	Description of the results: direct/indirect somatic embryogenesis
1.	M40 IAA 2.0 mg/l+BAP 0.5 mg/l	Direct somatic embryogenesis. The scales fold; embryos form inside the folded scales. Embryos formed after the first subculture on the same nutrient medium.
2.	M42 IAA 2.0 mg/l + BAP 2.0 mg/l	
3.	M50 IAA 1.0 mg/l +Kin 2.0 mg/l	
4.	M56 2.4D 0.5 мг/л + Кин 0.5 мг/л	Indirect somatic embryogenesis. On many explants, callus development is observed, followed by the formation of globular-shaped embryos. Indirect somatic embryogenesis. The formation of globular-shaped embryos is observed on many explants.
**№**	**Nutrient madia by Vollosovich (1979). Combination of phytohormones**	**Description of the results: direct somatic embryogenesis.**
1.	V68 IAA 0.5 mg/l + Kin 0.5 mg/l	The scales fold and embryos form inside of the folded scales. Embryos were formed after the first passage on the same nutrient medium. All formed embryos were characterized by a dense structure and globular phase. Basically, embryo formation takes place at the 3-5 subcultures.
2.	V68 IAA 0.5 mg/l + Kin 0.5 mg/l<br>V56 2.4 Д 0.5 mg/l +Kin 0.5 mg/l	
3.	V42.4D 0.5 mg/l + BAP 0.5 mg/l	
4.	V16 IAA 0.5 mg/l	
5.	V81 2.4 Д 0.5 mg/l + Kin 5.0 mg/l	
6.	V83 IAA 0.5 mg/l + BAP 5.0 mg/l	
7.	V84 2.4 Д 0.5 mg/l + BAP 5.0 mg/l	
8.	V87 2.4 Д 0.5 mg/l + Zea 5.0 mg/l	
9.	VK 1.0 Kin 1.0 mg/l	

Table S4 – Callusogenesis and direct somatic embryogenesis of the *Ungernia victoris*. Source of explants: bulb scales.

№	Nutrient media by Murashige and Skoog (1962) Combination of phytohirmones	Description of the results: callusogenesis
1.	M14 2.4D 2.0 mg/l	Week callusogenesis is observed.
2.	M15 2.4D 2.0 mg/l + BAP 1.0 mg/l	
3.	M16 2.4D 2.0 mg/l + BAP 2.0 mg/l	
4.	M18 2.4D 2.0 mg/l + BAP 4.0 mg/l	
5.	M20 2.4D 3.0 mg/l + BAP 0.5 mg/l	
6.	M23 2.4D 3.0 mg/l + BAP 3.0 mg/l	
7.	M24 2.4D 3.0 mg/l + BAP 4.0 mg/l	
8.	M26 2.4D 4.0 mg/l + BAP 0.5 mg/l	
**№**	**Nutrient madia by Vollosovich (1979). Combination of phytohirmones**	**The description of the results: direct somatic embryogenesis**
1.	V16 2.4D 2.0 mg/l + BAP 2.0 mg/l	The scales fold. and embryos form inside the folded scales. Embryos were formed after the first subculture on the same nutrient medium. All formed embryos were characterized by a dense structure, globular phase. Basically, embryo formation takes place at 3-5 subcultures.
2.	V4 2.4D 0.5 mg/l + BAP 2.0 mg/l	
**№**	**Nutrient madia by Vollosovich (1979). Combination of phytohirmones.**	**Description of the results: callusogenesis**
1.	V56 2.4D 0.5 mg/l + Kin 0.5 mg/l	The formation of dense calluses, yellow in color, is observed, mainly on the basal part of the explants.
2.	V5 2.4D 0.5 mg/l + BAP 3.0 mg/l	
3.	V44 2.4D 0.5 mg/l + Kin 2.0 mg/l	

Table S5 - Somatic embryogenesis on the nutrient medium of Murashige and Skoog (1962), %. Source of explants: bulb scales.

**Nutrient media**	***U. sewertzowii***	***U. victoris***
M40 IAA 2.0 mg/l + BAP 0.5 mg/l	60±2	5±0.2
M42 IAA 2.0 mg/l + BAP 2.0 mg/l	40±1.8	5±0.12
M50 IAA 1.0 mg/l + Kin 2.0 mg/l	60±1.5	5±0.21
M56 2.4D 0.5 mg/l + Kin 0.5 mg/l	50±1.8	5±0.18

Table S6 – The share (%) of the explants with the somatic embryogenesis on nutrient medium by Vollosovich et al. (1979). Source of explants: bulb scales.

**Nutrient media**	***U. sewertzowii***	***U. victoris***
V29 Kin 1.0 mg/l	60±2	5±2
V68 IAA 0.5 mg/l + Kin 0.5 mg/l	60±2	5±2
V68 IAA 0.5 mg/l + Kin 0.5 mg/l	10±2	3±2
V56 2.4D 0.5 mg/l + Kin 0.5 mg/l	15±2	3±2
V4 2.4D 0.5 mg/l + BAP 0.5 mg/l	20±2	10±2
V16 IAA 0.5 mg/l	15±2	5±2
V81 2.4D 0.5 mg/l + Kin 5.0 mg/l	20±2	2±2
V83 IAA 0.5 mg/l + BAP 5.0 mg/l	20±2	2±2
V84 2.4D 0.5 mg/l + BAP 5.0 mg/l	20±2	2±2
V87 2.4D 0.5 mg/l + Zea 5.0 mg/l	10±0.2	5±2
V16 2.4D 2.0 mg/l + BAP 2.0 mg/l	2±0.2	60±2
V4 2.4D 0.5 mg/l + BAP 2.0 mg/l	2±0.2	60±2

Table S7 – The share of the explants (%) with callusogenesis on the nutrient medium by Vollosovich (1979). Source of explants: bulb scales.

**Nutrient media**	***U. sewertzowii***	***U. victoris***
V68 IAA 0.5 mg/l + Kin 0.5 mg/l	60±1.9	0
V17 IAA 0.5 mg/l + BAP 0.5 mg/l	60±1.7	0
V56 2.4D 0.5 mg/l + Kin 0.5 mg/l	60±1.8	60±1.9
V5 2.4D 0.5 mg/l + BAP 3.0 mg/l	0	60±2
V44 2.4D 0.5 mg/l + Kin 2.0 mg/l	0	60±1.8

Table S8 – The share of the explants (%) with callusogenesis on the nutrient medium by Murashige and Skoog (1962). Source of explants: bulb scales.

**Питательная среда**	***U. sewertzowii***	***U. victoris***
M39 IAA 2.0 mg/l	10±2	0
M55 2.4D 0.5 mg/l	15±2	0
M60 2.4D 1.0 mg/l + Kin 0.5 mg/l	10±2	0
M56 2.4D 0.5 mg/l + Kin 0.5 mg/l	60±2	0
M14 2.4D 2.0 mg/l	2±2	10
M15 2.4D 2.0 mg/l + BAP 1.0 mg/l	2±2	15±2
M16 2.4D 2.0 mg/l + BAP 2.0 mg/l	3±2	15±2
M18 2.4D 2.0 mg/l + BAP 4.0 mg/l	4±2	18±2
M20 2.4D 3.0 mg/l + BAP 0.5 mg/l	3±2	5±2
M23 2.4D 3.0 mg/l + BAP 3.0 mg/l	4±2	10±2
M24 2.4D 3.0 mg/l + BAP 4.0 mg/l	3±2	15±2
M26 2.4D 4.0 mg/l + BAP 0.5 mg/l	2±2	15±2

Table S9 – *Ungernia victoris*. The induction of the callusogenesis on nutrient media with different combinations of phytohormones. Source of explants – segments of the germinated seeds (hypocotyl, cotyledon, radicle)

#	The name of nutrient media	The number of explants with callusogenesis (%)
1	M56 2.4Д 0.5 mg/l + Kin 0.5 mg/l	70±2
2	M5 2.4Д 0.5 mg/l + BAP 0.5 mg/l	65±1.2
3	M162 BAP 0.5 mg/l	65±2.2
4	M56 2.4Д 0.5 mg/l+Kin 0.5 mg/l+TDZ 0.5 mg/l	80±1.2
5	M5 2.4Д 0.5 mg/l+BAP 0.5 mg/l+TDZ 0.5 mg/l	80±1.2
6	M162 BAP 0.5 mg/l + TDZ 0.5 mg/l	60±2

Table S10 – *Ungernia victoris*. Indirect organogenesis on the nutrient medium by Murashige and Skoog (1962) with various combinations of the phytohormones. Source of explants: segments of the germinated seeds (hypocotyl, cotyledon, and radicle).

**Nutrient media**	**Number of microbulbs per explant during indirect organogenesis**
M44 NAA0.5 mg/l + Kin 0.5 mg/l	100-120
M68 IAA 0.5 mg/l + Kin 0.5 mg/l	100-150
M32 NAA 0.5 mg/l + BAP 0.5 mg/l	100-150
M17 IAA 0.5 mg/l + BAP 0.5 mg/l	1-2

Table S11 - Yield of extracts and antioxidant activity (DPPH and ABTS radical scavenging assays) in *Ungernia sewertzowii* (US) extracts.

**No.**	**Code**	**Place of collection**	**Yeild of Extracts**	**Antioxidant activity (Radical scavenging activity)***	
				**DPPH**	**ABTS**
			**[%]**	**IC_{50} [μg/mL]**	
***Ungernia sewertzowii* (Regel) B.Fedtsch. (Amaryllidaceae J.St.Hil.)**					
**Plant material collected from nature**					
25	US_1_1	Aksay, Tashkent region.<br>Collected in 2023.	14.82	1,157.05	594.25
26	US_1_2	Aksay, Tashkent region.<br>Collected in 2023.	(5)8.77	1,153.21	486.07
27	US_1_3	Aksay, Tashkent region.<br>Collected in 2023.	(2)3.29	566.88	458.05
28	US_2_1	Gulkamsay, Tashkent region.<br>Collected in 2023.	13.21	1,016.72	434.09
29	US_2_2	Gulkamsay, Tashkent region.<br>Collected in 2023.	14.52	1,000.10	549.79
30	US_2_1	Gulkamsay, Tashkent region.<br>Collected in 2023.	13.98	1,015.12	499.46
31	US_2_2	Gulkamsay, Tashkent region.<br>Collected in 2023.	13.03	994.37	618.47
32	US_3_1	Aksarsay, Tashkent region.<br>Collected in 2023	2.94	560.34	361.79
33	US_3_2	Aksarsay, Tashkent region.<br>Collected in 2023	6.34	622.00	300.52
34	US_4_1	Bildirsay, Tashkent region.<br>Collected in 2023	9.39	433.10	359.07
35	US_4_2	Bildirsay, Tashkent region.<br>Collected in 2023	7.74	442.00	335.62
***In vitro* regenerated plant material (leaves)**					
36	US_5	*in vitro* (US Bildirsay).<br>The plantlets, which developed in *in vitro*conditions	10.16	1,115.25	835.66
***In vitro* regenerated plant material (bulbs)**					
37	US_1_B	Aksay, Tashkent region.<br>Collected in 2023.	4.81	2,516.50	681.43
38	US_2_B	Gulkamsay, Tashkent region.<br>Collected in 2023.	3.33	5,346.56	1,t379.67
39	US_3_B	Aksarsay, Tashkent region.<br>Collected in 2023	7.37	ND	ND
40	US_4_B	Bildirsay, Tashkent region.<br>Collected in 2023	4.44	ND	ND
**Plant material collected from Botanical Garden**					
41	US_6	Botanical Garden.<br>Aksay, Tashkent region.<br>Collected in 2021.	(3)6.3	1,241.16	492.65
42	US_7	Botanical Garden<br>Gulkamsay, Tashkent region.<br>Collected in 2021.	6.81	1,305.33	508.61
43	US_8	Botanical Garden<br>Aksarsay, Tashkent region.<br>Collected in 2021	3.43	428.80	297.02
44	US_9	Botanical Garden<br>Bildirsay, Tashkent region<br>Collected in 2021	C 5.67	698.55	457.37
45	US_10	Botanical Garden<br>Gulkamsay, Tashkent region<br>Collected in 2021	9.23	1,058.81	357.21
**Callus**					
46	US3_V5	Vollosovich medium, 2.4D 0.5+BAP 0.5	(6)15.49	1,796.60	139.60
47	US3_V5+TDZ 0.5	Vollosovich medium 2.4D 0.5+BAP 0.5 +TDZ 0.5	(5)14.66	4,301.23	387.19

48	US3_M56	Murasige and Scoge medium, 2.4D 0.5 + Kin 0.5	12.45	3,131.77	222.91
49	US3_VK1.0	Vollosovich medium, Kin 1.0	15.89	1,267.62	39.18
50	US3_VK 1.0+2.4D 1.0	Vollosovich medium, Kin 1.0+2.4D 1.0	21.9	3,162.03	721.75
51	US3_V56	Vollosovich medium, 2.4D 0.5 + Kin 0.5	13.41	1,002.50	5.35
52	US1_V56	Vollosovich medium, 2.4D 0.5 + Kin 0.5	17.08	2,977.06	359.93
53	US3_VK1.0 (10.04)	Vollosovich medium, Kin 1.0	15.91	857.65	0.42
54	US3_M40	Murasige and Scoge medium, NAA 2.0+BAP 0.5	28.79	2,953.43	395.60
55	US3_V68	Vollosovich medium, IAA 0.5+KIN 0.5	11.36	3,785.91	164.32
56	US3_V57	Vollosovich medium, 2.4D+Kin 1.0	13.04	3,833.24	343.39
**Seeds**					
57	US1	Aksay, Tashkent region. Collected in 2023.	6.36	824.22	506.30

* Positive control: IC_{50} of Ascorbic acid DPPH 17.21 µg/mL, ABTS 8.33 µg/mL, respectively.

ND: non detected

Table S12 - Yield of extracts and antioxidant activity (DPPH and ABTS radical scavenging assays) in *Ungernia victoris* extracts.

No.	Code	Place of collection	Yeild of Extracts	Antioxidant activity (Radical scavenging activity)*	
				DPPH	ABTS
			[%]	IC_{50} [μg/mL]	
***Ungernia victoris* Vved. ex Artjush. (UV) (Amaryllidaceae J.St.Hil.)**					
**Plant material collected from nature**					
1	UV_1	Sangardak. Polgasay. Surkhandarya region<br>Collected in 10.04.2023.	10.5	2,397.77	751.30
2	UV_2	Nilu, Surkhandarya region<br>Collected in 15.04.2023.	7.67	1,367.95	533.82
3	UV_3	Pojaz, Surkhandarya region<br>Collected in 17.04.2023.	7.76	1,992.43	716.23
4	UV_4	Sovuqbuloq, Surkhandarya region<br>Collected in 17.04.2023.	3.08	1,803.88	471.23
***In vitro* regenerated plant material (leaves)**					
5	UV_5	*in vitro* (UV Sovuqbuloq). The plants developed in *in vitro* conditions.	8.58	1,542.79	823.83
***In vitro* regenerated plant material (bulbs)**					
6	UV_1_B	Sangardak. Polgasay. Surkhandarya region<br>Collected in 10.04.2023.	1.25	ND	ND
7	UV_2_B	Nilu, Surkhandarya region<br>Collected in 15.04.2023.	7.18	8,989.00	1,580.19
8	UV_3_B	Pojaz, Surkhandarya region<br>Collected in 17.04.2023.	5.34	4,409.07	1,496.65
9	UV_4_B	Sovuqbuloq, Surkhandarya region<br>Collected in 17.04.2023.	7.07	3,556.05	1,081.04
**Plant material collected from Botanical Garden**					
10	UV_6	Sovuqbuloq, Surkhandarya region<br>Collected from nature in 04.2022.	4.27	1,580.47	538.95
11	UV_7	Botanical Garden<br>The plants were collected in 2021 from Sangardak. Polgasay. Surkhandarya region.	6.3	2,389.94	591.30
12	UV_8	Botanical Garden<br>The plants were collected in 2021 from Nilu, Surkhandarya region	10.96	1,299.33	537.34
13	UV_9	Botanical Garden<br>The plants were collected in 2021 from Pojaz, Surkhandarya region	9.77	685.70	490.03
14	UV_10	Botanical Garden<br>The plants were collected in 2021 from Sovuqbuloq, Surkhandarya region.	9.02	923.46	603.82
15	UV_10-1	Botanical Garden<br>The plants were collected in 2021 from Sovuqbuloq, Surkhandarya region.	15.45	2,297.72	729.71
**Callus**					
16	UV1_V5+TDZ 0.5	Vollosovich medium 2.4D 0.5+BAP 0.5	8.41	955.09	2.15
17	UV1_VK 1.0+2.4D 1.0	Vollosovich medium, Kin 1.0+2.4D 1.0	10.14	1,142.16	31.38
18	UV_M56	Murasige and Scoge medium, 2.4D 0.5 + Kin 0.5	6.62	944.75	53.53
19	UV1_V57	Vollosovich medium, 2.4D+Kin 1.0	13.08	1,465.51	11.33
20	UV_V5	Vollosovich medium, 2.4D 0.5+BAP 0.5	15.22	825.57	4.91

21	UV1_V56	Vollosovich medium, 2.4D 0.5 + Kin 0.5	18.79	782.59	1.84
22	UV_V16	Murasige and Scoge medium, IAA 0.5	11.36	1,585.19	25.35
**Seeds**					
23	UV1	Sovuqbuloq, Surkhandarya region<br>Collected in 17.04.2023	16.23	3,595.11	3,538.89
24	UV2	Gissar range, Sangardak river, Sangardak village	14.75	ND	4,413.31

* Positive control: IC_{50} of Ascorbic acid DPPH 17.21 µg/mL, ABTS 8.33 µg/mL, respectively

ND: non detected
